# The implications of noncompliance for randomized trials with partial nesting due to group treatment

**DOI:** 10.1002/sim.8778

**Published:** 2020-10-28

**Authors:** Chris Roberts

**Affiliations:** ^1^ Centre for Biostatistics, Division of Population Health, School of Health Sciences University of Manchester Manchester UK

**Keywords:** group therapy, noncompliance, partially nested trials, therapist effect, treatment related clustering

## Abstract

Analyses of trials of group administered treatments require an identifier for therapy group to account for clustering by group. All patients randomized to receive the group administered treatment could be assigned an intended group identifier following randomization. Alternatively, an actual group could be based on those patients that comply with group therapy. We investigate the implications for intention‐to‐treat (ITT) analyses of using either the intended or actual group to adjust for the clustering effect. We also consider causal models using the actual group. A simulation study showed that ITT estimates based on random effects models or GEE with an exchangeable correlation matrix performed much better when using the intended group than the actual group. OLS with robust standard errors performed well with both. Most compliance average causal effect (CACE) models performed well. While practical constraints of the clinical setting may determine the choice between an intended or actual group analyses, it is desirable to record both. An ITT analysis using mixed models can then be fitted using the intended group with data generation assumptions checked by a causal model using the actual group. Where an ITT analysis is based on the actual group, worse outcome for never‐takers than compliers may allow one to infer that some estimators are biased toward no treatment effect. The work here is motivated and illustrated by a trial of a group therapy, but also has relevance to trials with treatment related clustering due to therapist examples of which include physical and talking therapies or surgery.

## 1 INTRODUCTION

Where treatment is administered to patients in groups rather than individually, for example, exercise classes for the treatment of musculo‐skeletal disorders,^1^ group therapies for psychological problems,^2^, ^3^ and self‐help groups for smoking cessation^4^ or alcohol problems,^5^ outcome for patients in the same group may correlated due to shared experience of delivery and interaction between patients.^6^ Ignoring the possible clustering effect of group treatment has been recognized as being statistically nonconservative as standard errors may be too small. Design and analysis of trials of group therapies should therefore assume that between‐therapy group variation in outcome is possible, which has implications for design and statistical analysis.^7^, ^8^ Similar clustering of patient outcomes by health professional may occur for talking, physical or surgical therapies, where factors such as experience or caseload may result in variation between healthcare professionals^9^ sometimes called the *therapist effect*. Such trials generally use individual randomization, so the clustering effect is due to the delivery of the treatment, not the method of randomization.^10^ As the clustering effect derives from treatment, it may differ between trial arms. This paper examines the implications of noncompliance in this setting.

The motivating example for this work is a trial of a group administered intervention for lower back‐pain.^11^ Patients were randomized to a new group cognitive behavioral therapy or control. Patients in the control arm received an educational pack for self‐management of back pain that was also given to group therapy patients. Clustering applied to just one randomized treatment arm so this trial had a partially nested design.^12^, ^13^

In this article, we assume that compliance is binary with patients either receiving or not receiving group therapy. Based on this assumption, one can identify four latent classes of patients: (i) *never‐takers*, that is, patients who do not receive the intervention irrespective of randomization, (ii) *always‐takers*, who always receive the group intervention irrespective of randomization, (iii) *compliers* who always adhere to the randomly allocated treatment, and (iv) *defiers* who always receive the opposite of their random allocation. First, we assume that there are no *defiers* whose exclusion is referred to as the *monotonicity assumption* in causal inference.^14^ Knowledge of compliance means that *never‐takers* can be identified in the intervention arm, but not in the control arm, and *always‐takers* can be identified in the control arm, but not in the intervention arm. We will also assume that randomization can only influence patient outcome by the receipt of the group treatment, an assumption referred to as the *exclusion restriction*. This assumption means that the outcome for *never‐takers* in the control arm is the same as that for *never‐takers* in the intervention arm, because they never receive the intervention. Similarly, the outcome for *always‐takers* is the same in both arms as they always receive the intervention. In the motivating example, the new group therapy was only available to patients randomized to that intervention, so there were no *always‐takers*.

Analyses of group therapies that take account of clustering require an identifier for the therapy group.^1^ One option would be the *intended* group, assigned for all patients randomized to group therapy. To guarantee that this is complete, it would need to be assigned at randomization. Let *IG*(*i*) be the identifier of the intended therapy group for the *i*th patient in the intervention arm. For a partially nested design, we require *IG*(*i*) to identify all patients in the control arm using a unique identifier for each patient. If there is noncompliance, some patients randomized to group treatment will not receive group treatment and so will not share the group experience. If we believe clustering is due to treatment, their outcome should not correlate with others assigned to the same group. Where compliance status is known, we can define *actual* group, say *AG*(*i*), identifying the therapy group for patients that adheres to group treatment. For all other patients, that is those in the control arm and all patients in the intervention arm that do not adhere to group treatment, *AG*(*i*) should be a unique identifier.

When the lower back pain trial was being planned, it was decided that a new therapy group would be initiated once sufficient numbers of patients had been randomized to the group intervention at a trial site. The particular group in which a patient received their treatment was recorded as each therapy group was initiated, but some patients randomized to the group therapy declined group treatment and so were not assigned to a therapy group. The identifier *AG*(*i*) was recorded, but not *IG*(*i*). In other trials, where *IG*(*i*) and compliance is recorded as a binary variable, *AG*(*i*) could be constructed retrospectively.

This article considers the following four questions:

1. How do methods for estimating intention‐to‐treat (ITT) effects using actual group *AG*(*i*) perform?
2. How do methods for estimating ITT effects using intended group *IG*(*i*) perform?
3. Where both *IG*(*i*) and *AG*(*i*) are recorded, which should be used for estimating ITT effects?
4. How do methods for estimating the compliance average causal effect (CACE) perform?

In Section 2, we present statistical methods for estimating ITT effects using either the intended or actual group. We then consider possible limitations of actual and intended group analyses. In Section 3, we will present methods for estimating the CACE introducing a new model for partially nested data utilizing a normal mixture distribution. In Section 4, simulation will be used to investigate the performance of methods listed in Sections 2 and 3. In Section 5, we apply these methods to data from the lower back pain trial that motivated this work. Finally, in Section 6, we discuss some of the implications of this work for design and analysis of this type of trial. Consistent with the motivating example, the work focuses on the situation where there are only two latent compliance classes, *compliers* and *never‐takers*. We consider the situation where there are also *always‐takers* in the discussion and Appendix A.

## 2 INTENTION TO TREAT ANALYSES FOR GROUP THERAPY TRIALS WITH NONCOMPLIANCE

### 2.1 Analysis methods

Consider a continuous outcome measure *y*_*i*_ for the *i*th patient. Suppose that *r*_*i*_ is an indicator variable for random assignment to group therapy. Where *r*_*i*_ = 0, *IG*(*i*) is a unique identifier of each subject. Define the indicator variable *t*_*i*_ equal to one if a patient adheres to the group intervention and zero otherwise. If there are no *always‐takers*, *t*_*i*_ = 0 in the control arm. Where *t*_*i*_ = 0, *AG*(*i*) is a unique identifier of each subject.

Suppose *δ* is the ITT effect. A simple analysis that takes no account of the clustering due to therapy group would be to fit the OLS model as follows,

(1) $$y_{i}=\alpha_{i}+{\delta r}_{i}+e_{i}^{\left(1\right)}$$

with $e_{i}^{\left(1\right)}∼N\left[0\sigma_{e}^{2}\right]$. The term *α*_*i*_ could be a constant or could depend on baseline covariates. Robust standard errors^15^ could be used to adjust for the effect of clustering using *IG*(*i*) for an intended group analysis or *AG*(*i*) for an actual group analysis. We refer to these methods as OLS(Rob‐Int) and OLS(Rob‐Act), respectively. An alternative would be to fit a GEE model^16^ with an exchangeable correlation matrix, again using robust standard errors with either intended or actual cluster that we refer to as GEE(Int) or GEE(Act).

One extension of Equation (1) is to fit a random intercept model using either the intended or actual group identifier as follows,

(2) $$y_{i}=\alpha_{i}+{\delta r}_{i}+u_{\mathrm{IG}\left(i\right)}^{\left(2\right)}+e_{i}^{\left(1\right)},$$

where $u_{\mathrm{IG}\left(i\right)}^{\left(2\right)}∼N\left[0\sigma_{u}^{2}\right]$ for an intended group analysis or

(3) $$y_{i}=\alpha_{i}+{\delta r}_{i}+u_{\mathrm{AG}\left(i\right)}^{\left(2\right)}+e_{i}^{\left(1\right)},$$

where $u_{\mathrm{AG}\left(i\right)}^{\left(2\right)}∼N\left[0\sigma_{u}^{2}\right]$ for an actual group analysis. We will refer to these methods as RI(Int) and RI(Act). Both models constrain the total variance to equal $\sigma_{u}^{2}+\sigma_{e}^{2}$ in both arms and the partitioning of the variance of subjects in the control arm is not identified. If the variance is misspecified, estimates of the random effect variances can be biased affecting test size.^7^ Despite these limitations, we will include both models in our simulation work below as they are sometimes used. To deal with the problem of variance misspecification in the control arm, Equations (2) and (3) can be replaced by

(4) $$y_{i}=\alpha_{i}+\delta r_{i}+u_{\mathrm{IG}\left(i\right)}^{\left(2\right)}r_{i}+e_{0i}^{\left(1\right)}\left(1-r_{i}\right)+e_{1i}^{\left(1\right)}r_{i}$$

or

(5) $$y_{i}=\alpha_{i}+\delta r_{i}+u_{\mathrm{AG}\left(i\right)}^{\left(2\right)}t_{i}+e_{0i}^{\left(1\right)}\left(1-r_{i}\right)+e_{1i}^{\left(1\right)}r_{i},$$

with $e_{0i}^{\left(1\right)}$ and $e_{1i}^{\left(1\right)}$having variances $\sigma_{e0}^{2}$ and $\sigma_{e1}^{2}$. We will refer to these methods as RE(Int) and RE(Act). In Equation (5), the variance of *never‐takers* in the intervention arm is $\sigma_{e1}^{2}$ and the variance for subjects receiving group treatment is $\sigma_{u}^{2}+\sigma_{e1}^{2}$, thereby constraining group therapy subjects to have greater variance than *never‐takers* in the intervention arm, if $\sigma_{u}^{2}>0$. To deal with this limitation, the residual variance for *compliers* and *never‐takers* in the group intervention arm can be allowed to differ by the model

(6) $$y_{i}=\alpha_{i}+\delta r_{i}+u_{\mathrm{AG}\left(i\right)}^{\left(2\right)}t_{i}+e_{0i}^{\left(1\right)}\left(1-r_{i}\right)+e_{1i}^{\left(1\right)}t_{i}+e_{2i}^{\left(1\right)}r_{i}\left(1-t_{i}\right),$$

with $e_{0}^{\left(1\right)}$,$\;e_{1}^{\left(1\right)}$, and $e_{2}^{\left(1\right)}$ having variances $\sigma_{e0}^{2}$, $\sigma_{e1}^{2}$, and $\sigma_{e2}^{2}$. We will refer to this method as RE(Act‐Het).

### 2.2 Possible limitation of methods of analysis

To investigate possible limitations of these methods, we will consider a data generation model based on the two latent compliance classes, *compliers* and *never‐takers*. Let us suppose that the mean outcome for *compliers* in the control arm is *μ*. If *τ* is the CACE, the mean outcome for *compliers* in the intervention arm is *μ + τ*. As a consequence of the *exclusion restriction*, we assume that the mean outcome for *never‐takers* is *μ + γ* in both arms. The data generation model is therefore:

(7) $$\text{Never}-\text{taker in both arms}:y_{i}=\mu +\gamma +e_{\mathrm{Ni}}^{\left(1\right)}\text{with}\;e_{\mathrm{Ni}}^{\left(1\right)}∼N\left[0,\sigma_{N}^{2}\right]$$

(8) $$\text{Complier control}:y_{i}={\mu +e}_{\mathrm{Ci}}^{\left(1\right)}\text{with}\;e_{\mathrm{Ci}}^{\left(1\right)}∼N\left[0,\sigma_{C}^{2}\right]$$

(9) $$\text{Complier group therapy}:y_{i}=\mu +\tau +u_{\mathrm{AG}\left(i\right)}^{\left(2\right)}t_{i}+e_{\mathrm{Ti}}^{\left(1\right)}\text{with}\;e_{\mathrm{Ti}}^{\left(1\right)}∼N\left[0,\sigma_{T}^{2}\right]\;\text{and}\;u_{\mathrm{AG}\left(i\right)}^{\left(2\right)}∼N\left[0,\sigma_{u}^{2}\right].$$

The subscripts of error terms differ from Equation (6) as here they may relate to the variance of subjects in latent classes rather than observable subsets. Specifically, the random effect $e_{\mathrm{Ci}}^{\left(1\right)}$ in Equation (8) should not be confused with $e_{0i}^{\left(1\right)}$ in Equation (6) as the former is the residual term of compliant subjects in the control arm whereas the latter is the residual of all control arm subjects. Note also, $e_{\mathrm{Ni}}^{\left(1\right)}$ in Equation (7) is the residual term for *never‐takers* in both arms whereas $e_{2i}^{\left(1\right)}$ is only present in the intervention arm in Equation (6).

Suppose *π* is the proportion of subjects that comply. Assuming equal size therapy groups, the design effect is *D* = 1 + (*m* − 1)*ρ* where *m* is the group size and *ρ* is the ICC equal to $\sigma_{u}^{2}/\left(\sigma_{u}^{2}+\sigma_{T}^{2}\right)$. Analyses of clustered data using GEE with an exchangeable correlation matrix or random effects models weight subjects in clusters by 1/*D*. This is not an issue for intended group analyses, as all subjects in the group therapy arm are in clusters, but it is a concern for analyses based on actual group. *Never‐takers* in the intervention arm will not be down weighted by 1/*D* as they are not in clusters. In the control arm all subjects will have a weight of 1. Hence, *compliers* in the intervention arm make a smaller contribution to the intervention arm mean than *compliers* in the control arm to the control arm mean. This will bias ITT estimates based on an actual group analysis. In Appendix A, we derive an approximation for the ITT effect for an actual group analysis as:

(10) $$\delta_{w}=\pi \left(\frac{\;\tau +\gamma \left(1-\pi \right)\left(D-1\right)}{\pi +\left(1-\pi \right)D}\right).$$

If *ρ* = 0, the design effect *D* = 1 and the approximation simplifies to *πτ*, which is a standard result for the relationship between ITT and CACE effects for trials without clustering. Where *ρ* > 0, *δ*_*w*_ ≠ *πτ*, except where *γ* = *τ*. The bias of an actual group estimate of the ITT treatment effect can be approximated by:

(11) $$\left(\gamma -\tau \right)\left(\frac{\pi \left(1-\pi \right)\left(D-1\right)}{\pi +\left(1-\pi \right)D}\right).$$

This suggests that an estimate of the treatment effect using an actual group analysis could be biased, if the mean outcome of *never‐takers* (*μ* + *γ*) differs from the mean outcome of *compliers* in the intervention arm (*μ* + *τ*). Expression (11) implies that the bias is a linear function of *γ* equal to zero when *γ* = *τ*. Where *γ* > *τ* the bias is positive, and where *γ* < *τ* the bias is negative.

Use of actual group also biases the estimation of the random effect variance, $\sigma_{u}^{2}$. In the data generation model patients receiving group treatment have a mean *μ + τ* with $\sigma_{u}^{2}$ being the variance about that mean. Equations (3), (5), and (6) estimate $\sigma_{u}^{2}$ about the mean or adjusted mean of the intervention arm. Unless *γ* = *τ*, the estimates of $\sigma_{u}^{2}$ from Equations (3), (5), and (6) will be inflated by the square of the difference between the mean of compliers and the mean of the intervention arm. In Appendix A, we derive the following approximation for the estimate of ICC for Equations (3) and (5) as:

(12) $$\left(\frac{\rho +{\left(\frac{\left(\gamma -\tau \right)\left(1-\pi \right)D}{\left(\pi +\left(1-\pi \right)D\right)}\right)}^{2}}{1+{\left(\frac{\left(\gamma -\tau \right)\left(1-\pi \right)D}{\left(\pi +\left(1-\pi \right)D\right)}\right)}^{2}}\right),$$

which is greater than *ρ*. Since expression (11) has a positive derivative with respect to *D*, an upward bias of the ICC will further accentuate the treatment effect bias. As |*γ* − *τ*| increases expression (11) will increasingly under‐estimate the magnitude of the treatment effect bias.

Equation (6) estimates separate variances, $\sigma_{e1}^{2}$ and $\sigma_{e2}^{2}$, for *compliers* and *never‐takers* in the group intervention arm. This introduces another source of bias as *never‐takers* in this arm will now be weighted in proportion to $1/\sigma_{e2}^{2}$. Expression (11) can be modified by replacing *D* by λ *D*, where $\lambda =\left(\sigma_{e1}^{2}+\sigma_{u}^{2}\right)/\sigma_{e2}^{2}$, to give an approximation for the bias of the treatment effect estimated by Equation (6) as:

(13) $$\left(\gamma -\tau \right)\left(\frac{\pi \left(1-\pi \right)\left(\lambda \;D-1\right)}{\pi +\left(1-\pi \right)\lambda D}\right).$$

Where λ > 1/*D*, the bias will be positive where *γ > τ* and will be negative where *γ* < *τ*. Where *λ* < 1/*D*, signs will reverse. An approximate estimate of the ICC can be obtained by replacing *D* by *λ* *D* in expression (12). The misspecification of the mean that biases $\sigma_{u}^{2}$ will also affect the residual variance of *never‐takers* in the intervention arm, $\sigma_{e2}^{2}$. In the data generation model, this residual term is about *μ* + *γ*, but $\sigma_{e2}^{2}$ is estimated about the mean or adjusted mean of the intervention arm so its value will increase as |*γ* − *τ*| increases. An estimate of *λ* could be biased where *γ* ≠ *τ* which in turn affects expression (13), which is also affected by *ρ* via the design effect *D*.

Expressions (11) and (13) are crude approximations for the possible bias of treatment effect estimates by an actual group analysis as they take no account of the bias of the ICC, and also *λ* for expression (13). Nevertheless, these expressions suggest that treatment effect estimates based on an actual group analysis, using either Equation (3), (5), or (6) could be biased, and also suggest factors that may affect the magnitude of these biases.

Consider now an intended group analysis. Since *never‐takers* do not receive the group experience, it seems unsatisfactory to associate *never‐takers* with the random effect for therapy group as happens in Equations (2) and (4). Inclusion of the unexposed *never‐takers* will dilute the clustering effect, so one would expect estimates of the clustering variance $\sigma_{u}^{2}$ and the ICC to be biased toward zero. While this will make the adjustment for clustering too small, it will be off‐set by the larger cluster size of intended group analyses. Equation (4) also constrains the variance of subjects in the intervention arm to be $\sigma_{u}^{2}+\sigma_{e1}^{2}$, which could further bias $\sigma_{u}^{2}$.^7^

## 3 CAUSAL METHODS FOR THE ANALYSIS OF A PARTIALLY NESTED DESIGN WITH NONCOMPLIANCE

There is a large body of literature that considers estimation of causal treatment effects in individually randomized trials where there is no clustering.^17^, ^18^ These have been extended to the estimation of causal effects in cluster randomized trial by Jo et al^19^ and Schochet and Chiang.^20^

Above we define the variable *t*_*i*_ that identifies those patients that receive treatment. In the current setting, with no *always‐takers*, this identifies compliance status in the intervention arm. A standard method that can be used to estimate the causal treatment effect is instrumental variables regression.^21^ In a comparison of methods of analysis of cluster randomized trials with noncompliance, Moerbeek and van Schie^22^ used instrumental variables with robust standard errors to control for the effects of clustering. This method^15^ can also be used to protect test size in this setting. An alternative would be the use of an instrumental variable model with a random effect^23^ for group therapy. We will refer to these methods as IV, IV(Rob), and RE‐IV, respectively.

A second method of estimation of the causal effect is the Bloom CACE estimator^24^ to which a random effect can be added. Define *s*_*i*_ equal to 1 for a *never‐taker* in the group therapy arm, and zero otherwise, so that *s*_*i*_ = (1 − *t*_*i*_)*r*_*i*_. For the *i*th subject

(14) $$y_{i}=\alpha_{i}+\beta_{t}t_{i}+\beta_{s}s_{i}+u_{\mathrm{AG}\left(i\right)}^{\left(2\right)}t_{i}+e_{0i}^{\left(1\right)}\left(1-r_{i}\right)+e_{1i}^{\left(1\right)}t_{i}+e_{2i}^{\left(1\right)}s_{i},$$

with $e_{0i}^{\left(1\right)}$,$\;e_{1i}^{\left(1\right)}$, and $e_{2i}^{\left(1\right)}$ having variances $\sigma_{e0}^{2}$, $\sigma_{e1}^{2}$, and $\sigma_{e2}^{2}$. The Bloom CACE estimate of the treatment effect is then $\left(\hat{\pi }\;{\hat{\beta }}_{t}+\left(1-\hat{\pi }\right){\hat{\beta }}_{s}\right)/\hat{\pi }$, where $\hat{\pi }$ is the estimate of the rate of compliance determined from the intervention arm. We will refer to this estimator as RE‐BLM.

Another method of estimation of causal effects in randomized trials is to use a normal mixture model to differentiate the latent classes of *compliers* and *never‐takers* in the control arm.^25^ Jo adapted this method for the analysis of cluster randomized trials.^19^, ^26^ We can use a similar approach here. For simplicity of presentation, we will give separate equations for the intervention and control arms. Where there is no clustering, the intervention arm model is

(15) $$y_{i}=\alpha_{i}+\tau t_{i}+\gamma \left(1-t_{i}\right)+e_{\mathrm{Ci}}^{\left(1\right)}t_{i}+e_{\mathrm{Ni}}^{\left(1\right)}\left(1-t_{i}\right),$$

with $e_{\mathrm{Ci}}∼N\left[0\sigma_{C}^{2}\right]$ and $e_{\mathrm{Ni}}∼N\left[0\sigma_{N}^{2}\right]$ and *t*_*i*_ is the indicator variable for compliance with a Bernoulli distribution *B*(*π*) where *π* is the compliance rate. For the control arm

(16) $$y_{i}=\alpha_{i}+\xi_{i}^{\left(1\right)},$$

where$\;\xi_{i}^{\left(1\right)}$is the normal mixture model $\xi_{i}^{\left(1\right)}∼N\left[0\sigma_{C}^{2}\right].\pi +N\left[\gamma \sigma_{N}^{2}\right].\left(1-\pi \right)$. We will refer to this as method MM. One way to account for clustering is to use robust standard errors, which we refer to as MM(Rob). Another approach would be to add a random effect to Equation (15) making the model for the intervention arm

(17) $$y_{i}=\alpha_{i}+\tau t_{i}+\gamma \left(1-t_{i}\right)+u_{\mathrm{AG}\left(i\right)}^{\left(2\right)}t_{i}+e_{\mathrm{Ci}}^{\left(1\right)}t_{i}+e_{\mathrm{Ni}}^{\left(1\right)}\left(1-t_{i}\right),$$

with $u_{\mathrm{AG}\left(i\right)}^{\left(2\right)}∼N\left[0\sigma_{u}^{2}\right]$, which will be referred to as RE‐MM.

The models presume the same variance for *compliers* in the control and treated arms, but treatment may change the variance of treated subjects. This heteroscedasticity could be modeled by adding a third level 1 variance term, say $e_{\mathrm{Ti}}∼N\left[0\sigma_{T}^{2}\right]$ to replace $e_{\mathrm{Ci}}^{\left(1\right)}$ in Equations (15) and (17). We will refer to these models as MM(Het), MM(Het‐Rob), and RE‐MM(Het).

Where clustering has been considered, the causal models above all use the actual group identifier, *AG*(*i*). While one could replace this by the intended group identifier *IG*(*i*), this does not correspond to the causal model of clustering define by Equation (9). Use of a random effects term $u_{\mathrm{IG}\left(i\right)}^{\left(2\right)}r_{i}$ in place of $u_{\mathrm{AG}\left(i\right)}^{\left(2\right)}t_{i}$, would not identify the variance components of *never‐takers* in the control arm. Given that the purpose of these models is to investigate the data generation model, we have not considered causal models with $u_{\mathrm{IG}\left(i\right)}^{\left(2\right)}r_{i}$ as they do not correspond to the causal pathway of the clustering effect.

## 4 SIMULATION STUDY TO EVALUATE ITT AND CAUSAL METHODS

### 4.1 Simulation study design

The objectives of the simulation study were to investigate possible bias due to noncompliance of estimates of (i) the treatment effect, (ii) the intra‐cluster correlation coefficient of group treatment, and (iii) the coverage of a 95% confidence interval of the methods listed in Sections 2 and 3. In the setting of full compliance, it has been shown that between arm heteroscedasticity may bias estimates of the intra‐cluster correlation, and hence coverage, and so it is recommended that this is modeled.^7^ Where there is noncompliance the *exclusion restriction* implies that the variances of *never‐takers* in intervention arm equals that of the control arm. Heteroscedasticity could occur between *never‐takers* and *compliers* and this could differ between treatment arms. Inclusion of heteroscedasticity between *never‐takers* and *compliers* in the control arm was felt to be of less interest than between *never‐takers* and *compliers* in the intervention arm as the latter is confounded with the clustering effect. Therefore, we set the variance of *never‐takers* in either arm and *compliers* in the control arm equal to 1. Above, we defined *λ* as the ratio of the variance of the compliers in the intervention arm to the variance of *never‐takers* in the control arm. Three values of *λ* were considered: equality of variance (*λ =* 1), reduced variance of complying subjects in the intervention arm (*λ =* 2/3), and increased variance of complying subjects (*λ =* 3/2).

Without loss of generality, we will specify the mean of compliant subjects in the control to be zero so that the mean of the *never‐takers* is *γ*_._ Data generation was based on the latent data structure using Equations (7), (8), and (9) with *μ* = 0, $\sigma_{N}^{2}=\sigma_{C}^{2}=1$, $\sigma_{T}^{2}=\left[0,\left(1-\rho \right)\lambda \right]$, and $\sigma_{u}^{2}=\rho \lambda$. Compliance class was determined by a Bernoulli distribution *B*(*π*) where *π* is the rate of compliance.

For the causal treatment effect estimates bias can be defined as the difference between the estimate and the data generating value of *τ*. For the ITT treatment effect bias can be defined as the difference between the estimate and *πτ*. Expressions (11) and (13) suggest that the treatment effect bias will be zero where *γ* equals*τ*. Different values of *τ* would simply move figures side‐ways on the scale of *γ*. To reduce the number of scenarios, the work presented here was restricted *τ* = 0 as we felt that extensive simulation work with other values of *τ* would add little.

To examine the effect of *γ* on bias and coverage, we chose nine values (*γ =* − 1, − 0.75, − 0.5, − 0.25, 0, 0.25, 0.5, 0.75, 1). We also considered an intra‐cluster correlation for group therapy (*ρ*) of 0.05 and 0.1 and compliance rate (*π*) of 80% and 70%. The actual group identifier *AG*(*i*) was constructed from the intended group *IG*(*i*) by replacing the group identifier with the patient identifier for noncompliant subjects. Constructed in this way actual therapy groups are nested in intended groups. We considered intended groups of size 5 or 10, which are plausible for a clinical group intervention. Hence, the expected actual group sizes were 4 and 8 for 80% compliance and 3.5 and 7 for 70% compliance. Where all members of a group were noncompliant, the number of clusters reduced by 1. Where just one members of a cluster complied, this subject was analyzed according to their compliance status as a cluster of size 1. Since the data generating model for that subject was the same as other compliant subjects in the intervention arm, this will not bias the estimate of $\sigma_{u}^{2}$.

In pilot work, we found that the simulation failure rate depended on sample size per arm. When a sample size of 100 subjects per arm was used, it gave a failure rate of 0.4%, whereas a sample size of 200 per arm gave a rate of 0.2%. We therefore used a sample size of 200 per arm to minimize possible bias due to simulation failures.

For each scenario, we carried out 10 000 simulations, which gives a power of greater than 90% to detect an absolute 1% change in the coverage of a 95% confidence interval. Simulation work, carried out using Stata version 15,^27^ took approximately 3 months using all 4 cores of a quad core Windows 10 PC. All methods were fitted using the standard Stata procedure except those based on mixture models, where bespoke algorithms using Stata's maximum likelihood procedure were written. The code for data generation and statistical analyses are given in Appendix 3 in Supporting Information. All methods of analysis are listed in Table 1 together with the Stata procedure name and the estimation equation where they are defined. It was assumed that compliance status was only observed in the intervention arm. All simulation work assumed outcome data was complete.

**TABLE 1:** Simulation study analysis methods

Method of analysis	Clustering	Stata procedure	Acronym	Equation number in text
Intention to treat				
Linear model	Ignored	regress	OLS	(1)
Linear model with robust standard errors	Intended /Actual	regress	OLS(Rob‐Act) / OLS(Rob‐Int)	(1)
GEE model with an exchangeable correlation matrix and robust standard errors	Intended /Actual	xtgee	GEE(Act)/ GEE(Int)	(1)
Random intercept model	Intended /Actual	xtreg	RI(Act) / RI(Int)	(2)/(3)
Random effects model with between arm heteroscedasticity	Actual /Intended	xtmixed	RE(Act) / RE(Int)	(4)/(5)
Random effects model with heteroscedasticity between arms and within intervention arms	Actual	xtmixed	RE(Act‐Het)	(6)
Causal models				
Instrumental variables regression with or without robust standard errors	Ignored / Actual	ivregress	IV, IV(Rob)	Not given. See Section 3
Instrumental variables regression with a random effect	Actual	xtivreg	RE‐IV
Bloom CACE estimator with random effects	Actual	xtmixed	RE‐BLM	(14)
Mixture model without /with robust standard errors	Ignored /Actual	bespoke Stata code	MM MM(Rob)	(15) and (16)
Mixture model with between arm heteroscedasticity^a^ without/ with robust standard errors	Ignored /Actual	bespoke Stata code	MM(Het) MM(Het‐Rob)	(15) and (16)
Mixture model with a random effect without /with heteroscedasticity^a^ for compliers	Actual	bespoke Stata code	RE‐MM / RE‐MM(Het)	(16) and (17)

^a^ To allow for heteroscedasticity $e_{\mathrm{Ci}}∼N\left[0\sigma_{C}^{2}\right]$ is replace by $e_{\mathrm{Ti}}∼N\left[0\sigma_{T}^{2}\right]$ in the intervention arm.

### 4.2 Actual group analyses: Intention‐to‐treat

#### 4.2.1 Treatment effect

**FIGURE 1:**
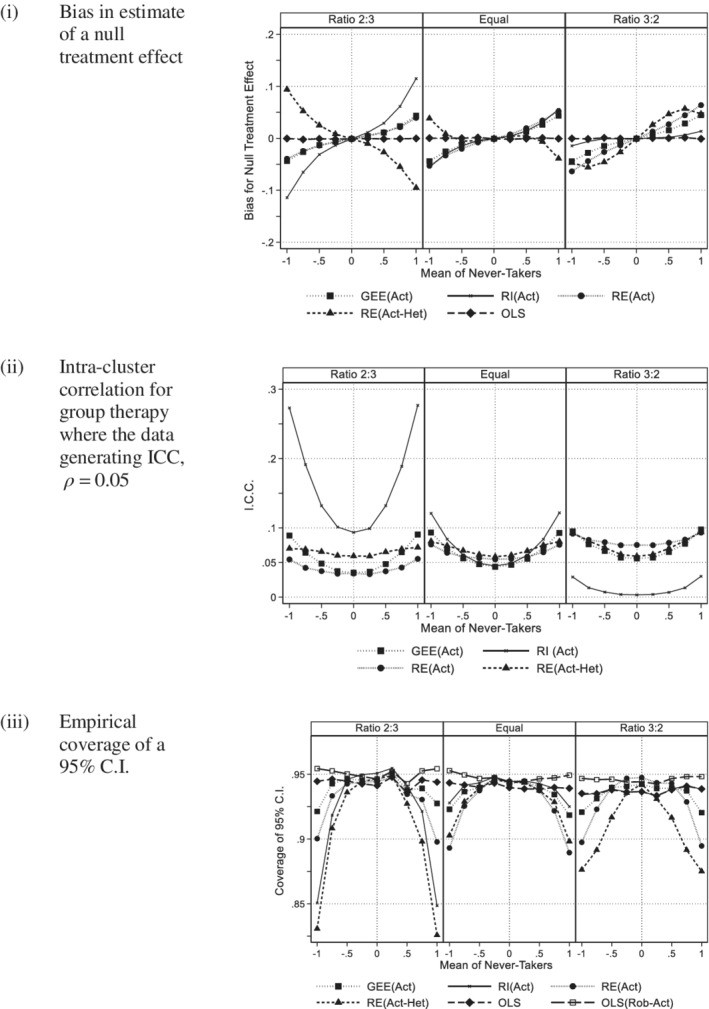
Actual group: 200 subjects per arm (Group size 5, ICC 0.05, 80% compliance with a panel per variance ratio). A, Bias in estimate of a null treatment effect. B, Intra‐cluster correlation for group therapy where the data generating ICC, *ρ =* 0.05. C, Empirical coverage of a 95% C.I

Figure 1 plots the simulation estimates against the mean of the *never‐takers* (*γ*) where *m* = 5, *ρ* = 0.05, *τ* = 0, and *π* = 0.8 with separate panels for each value of the variance ratio (*λ =* 2/3, 1, 3/2). Figure 1A gives the mean of estimates of the null treatment effect for four methods of analysis using actual group and the OLS estimate. OLS(Rob‐Act) has not been shown as the estimate are identical to OLS. As would be expected, OLS showed negligible bias as all subjects are weighted equally. The plots of estimates for GEE(Act), RI(Act), and RE(Act) all had a positive gradient against *γ*, which is consistent with expression (11). They also had negligible bias where *γ* = 0. When cluster size was increased to 10, (see Appendix 2, figure A1, in Supporting Information), the bias for GEE(Act), RI(Act), and RE(Act) all increased substantially. Increasing the ICC from 0.05 to 0.1 also increased the bias. In all cases, the plots were monotone increasing.

The plots of bias for RE(Act‐Het) also had negligible bias where *γ* = 0. Where *λ* = 2/3 the plots had either a negligible or negative gradient (figure A1), in contrast to GEE(Act), RI(Act), and RE(Act). Where *λ* = 1 or 3/2 and the cluster size was 5, the plot of bias was not monotone. For *λ* = 1 or 3/2 and a cluster size of 10, the bias was similar to that of GEE(Act), RI(Act), and RE(Act).

Appendix figure A2 compares the empirical bias of the treatment effect for RE(Act) and RE(Act‐Het) with the approximations given by expressions (11) and (13), for cluster sizes of 5 and 10 and intra‐cluster correlations of 0.05 and 0.1. The nonmonotone behavior of RE(Act‐Het) observed in Figure 1A and figure A1 can now be clearly seen in 4 of the 12 panels. Expressions (11) and (13) are labeled *Predicted RE(Act)* and *Predicted RE(Act‐Het)*, respectively. So that we can observe behavior close to *γ* = 0, we have allowed the vertical scale to change between figures. Expression (11) does not contain the variance ratio *λ* so it is the same in each panel of each figure, whereas the gradient of expression (13) increases as *λ* increases equaling expression (11) when *λ* = 1. Where |*γ*| was small, the lines define by expressions (11) and (13) were close to the empirical values, but as |γ| increased the predicted and observed values diverged. As suggested in Section 2.2 expression (11) will under‐estimate the bias and this was observed in figure A2, whereas expression (13) both over and under estimate the bias. Neither expression is a good predictor, but they give some insight into the cause of the treatment effect bias.

**FIGURE 2:**
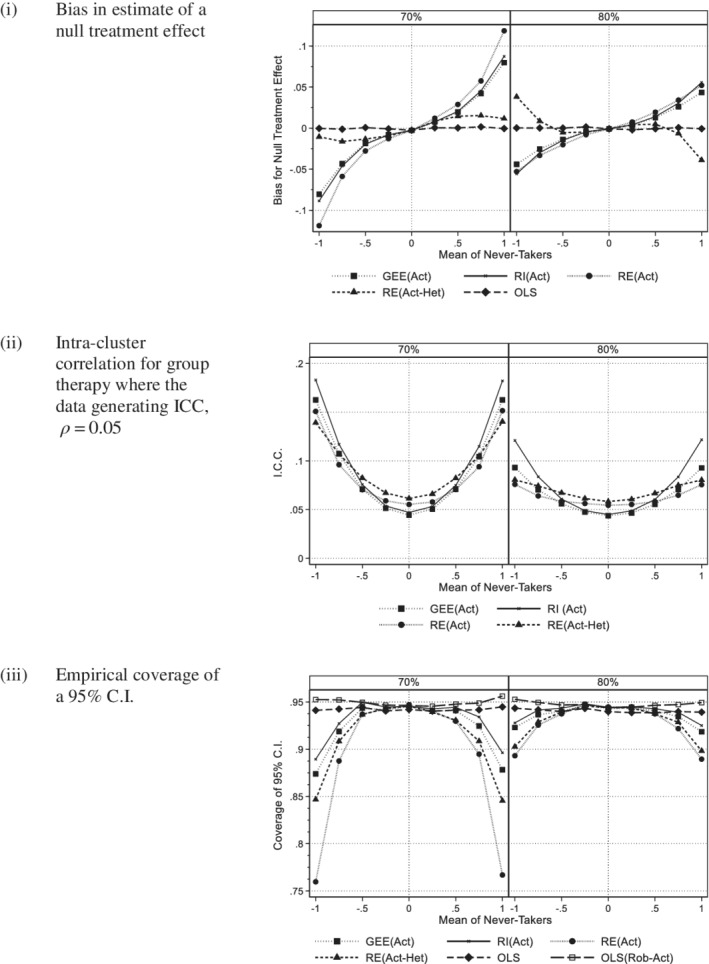
Actual group: Comparison of 70% and 80% compliance (200 subjects per arm, Group size 5, ICC 0.05, equal variance). A, Bias in estimate of a null treatment effect. B, Intra‐cluster correlation for group therapy where the data generating ICC, *ρ =* 0.05. C, Empirical coverage of a 95% C.I

Figure 2A compares the bias for 70% and 80% compliance. Reduction in the rate of compliance from 80% to 70% increased the gradient. For GEE(Act), RI(Act), and RE(Act), the magnitude of the bias increased, whereas for RE(Act‐Het) a negative bias was attenuated.

#### 4.2.2 Intra‐cluster correlation

Figure 1B illustrates the ICC for those methods that estimate this term. The plots have a “quadratic” shape as suggested by expression (12). For all methods, there was a change in the magnitude of the ICC as the variance ratio changed. This was particularly apparent for the random intercept model, RI(Act), which is expected as this method has been shown to be sensitive to between arm heteroscedasticity even with full compliance.^7^ When the cluster size was increased from 5 to 10 or when the data generating ICC increased from 0.05 to 0.1, the bias of the ICC was greater (see appendix figure A3). In Figure 2B, the bias was greater for 70% compliance than 80% for all methods.

#### 4.2.3 Coverage

Figure 1C shows the coverage of a 95% C.I. for all six methods. Those methods that showed bias in Figure 1A,B had reduced coverage indicating that the intervals were too narrow making the procedure nonconservative. This was particularly so for those including a random effect. OLS with robust SE based on the actual group had the correct coverage. Increasing cluster size from 5 to 10 or increasing the intra‐class correlation coefficient from 0.05 to 0.1 increased these effects (see appendix figure A4). Due to the effect of clustering, OLS became more nonconservative, whereas OLS with robust standard errors maintained satisfactory coverage. In Figure 2C, we see that reducing compliance from 80% to 70% gave worse coverage for the random effects methods and GEE, but did not affect OLS with robust standard errors.

### 4.3 Intended group analyses: Intention‐to‐treat

#### 4.3.1 Treatment effect

**FIGURE 3:**
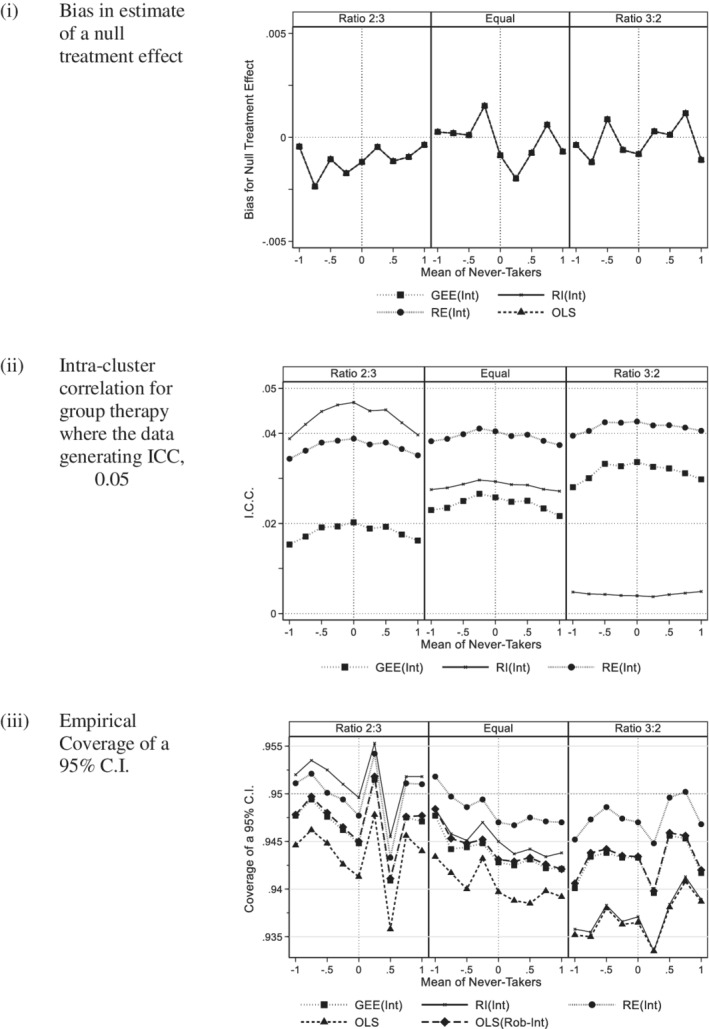
Intended group: 200 subjects per arm (Group size 5, ICC 0.05, 80% compliance with a panel per variance ratio). A, Bias in estimate of a null treatment effect. B, Intra‐cluster correlation for group therapy where the data generating ICC, *ρ =* 0.05. C, Empirical coverage of a 95% C.I

**FIGURE 4:**
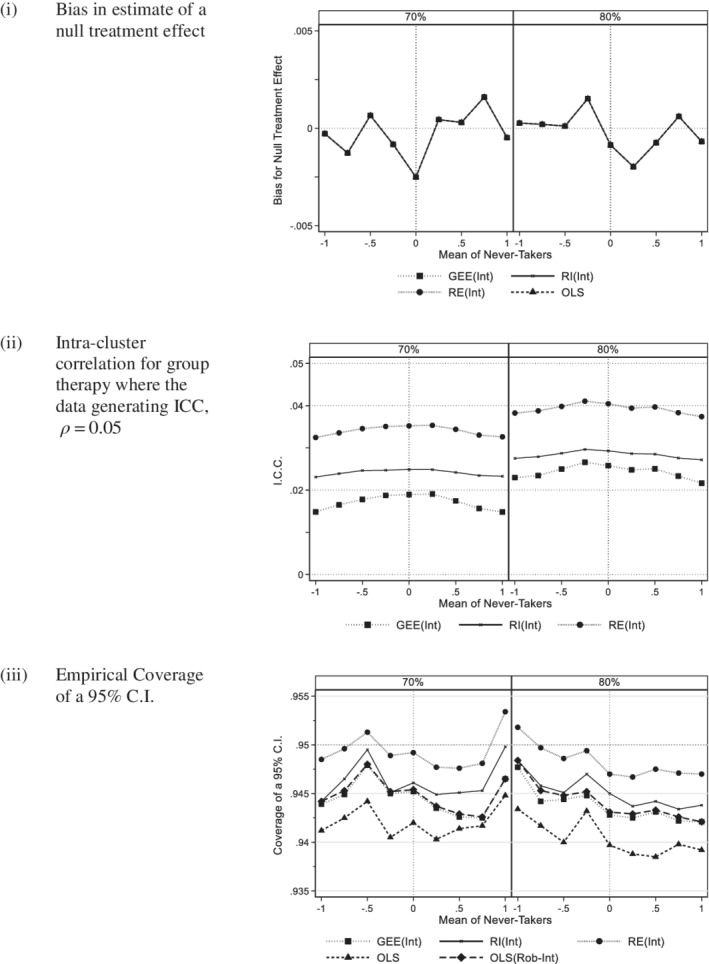
Intended group: Comparison of 70% and 80% compliance (200 subjects per arm, Group size 5, ICC 0.05, equal variance). A, Bias in estimate of a null treatment effect. B, Intra‐cluster correlation for group therapy where the data generating ICC, *ρ =* 0.05. C, Empirical coverage of a 95% C.I

Figure 3A shows the bias for the ITT effect for intended group methods. OLS(Rob‐Int) has not been plotted as it gives the same estimated of the treatment effect as OLS. The vertical scale is now very different to that for the actual group analyses in Figure 1A. All methods gave almost identical estimates of bias and these were much smaller. Had there been variation in intended group size we would expect there to be slightly larger differences between methods. Figure 4A compares a compliance rate of 70% with 80%. As with 80% compliance, there was no evidence of bias for 70% compliance.

#### 4.3.2 Intra‐cluster correlation

Figure 3B shows the intra‐cluster correlation of those methods that estimate this term. All values were reduced compared to the data generating value of 5%. This is to be expected as the clustering effect will be diluted by noncompliance. The RI(Int) and GEE(Int) estimates of the ICC were affected by heteroscedasticity whereas the RE(Int) estimates were more stable, which is consistent with the literature.^7^ Similar patterns were seen for larger cluster size and/or larger ICC (see appendix figure A6). Reducing compliance from 80% to 70% reduced the intra‐cluster correlation further (see Figure 4B).

#### 4.3.3 Coverage

In Figure 3C, coverage is considered. There was less evidence of bias than for actual group analyses with the lowest coverage occurring for OLS, and also the random intercept model, RI(Int) where the variance ratio is 3:2. Poor coverage of OLS is to be expected as it takes no account of clustering. The reduction in the coverage for RI(Int) can be explained by biased estimate of the ICC seen in Section 4.3.2. The random effects model RE(Int) gave coverage closest to the desired value and GEE and OLS with robust standard errors also performed well. The corresponding results for larger cluster size and/or larger ICC are given in appendix figure A7, where again RE(Int) was the best performing method, although coverage was slightly lower for a larger cluster size and/or a larger ICC. Reduction in compliance from 80% to 70% did not appear to affect coverage (Figure 4C).

### 4.4 Comparison of actual and intended group analyses

**FIGURE 5:**
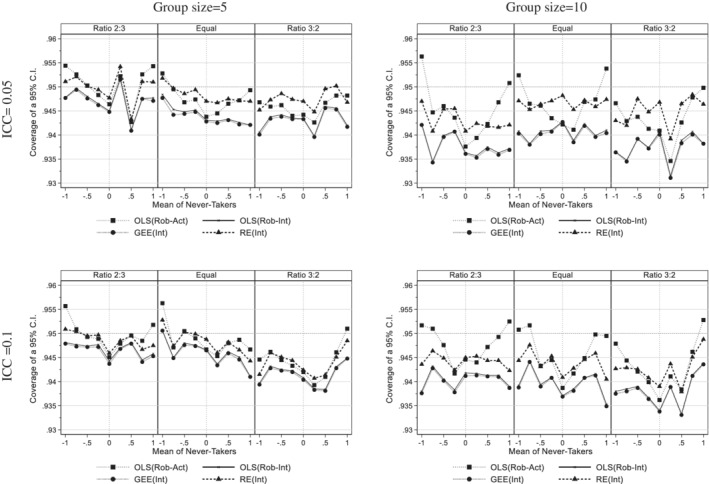
Comparison of 95% C.I. Coverage for Actual and Intended group methods (200 subjects per arm, 80% compliance and a panel per variance ratio)

For an actual group analysis, the only method with acceptable coverage across the range of parameter values tested was OLS(Act). For intended group analysis, a random effects model allowing for between arm heteroscedasticity (RE(Int)) performed best in terms of coverage. OLS and GEE with robust standard errors also performed well. The treatment effects estimates were the same for all four methods. Two of the four methods gave estimates of the ICC with RE(Int) giving better estimates than GEE(Int). In Figure 5, we compare coverage of all four methods. Coverage tended to be better for the smaller cluster size. Averaging across simulation scenarios, the mean coverage of 95% confidence intervals were 94.7% for OLS(Act), 94.6% for RE(Int), and 94.2% for OLS(Int) and GEE(Int).

### 4.5 Causal methods

#### 4.5.1 Treatment effect

**FIGURE 6:**
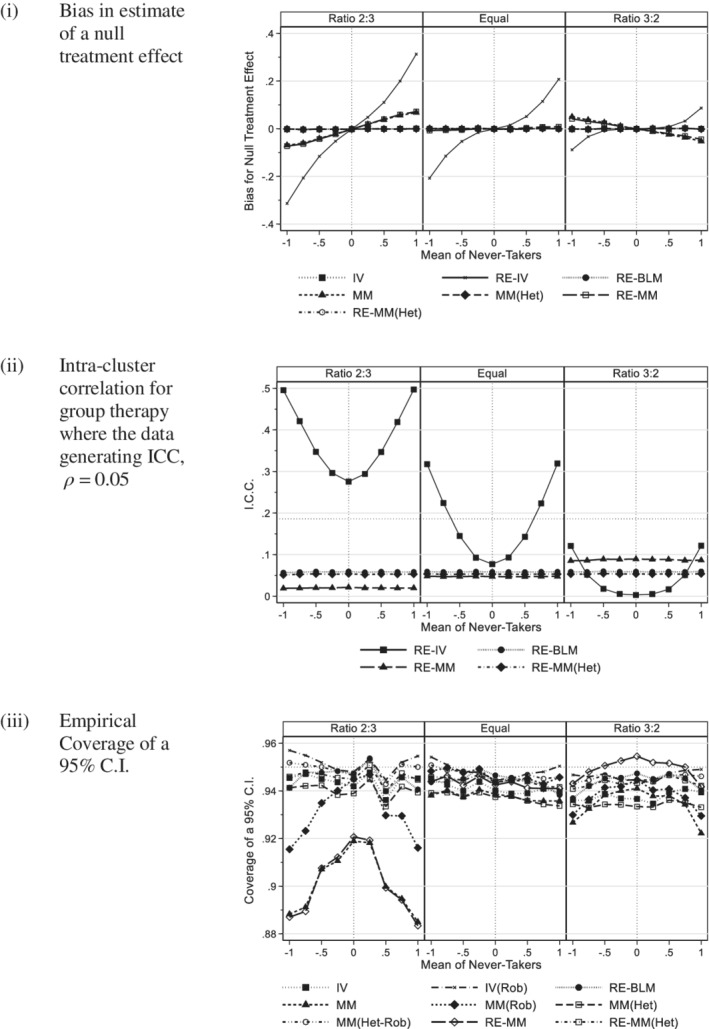
Causal models with actual group (200 subjects per arm, group size 5, ICC 0.05, 80% compliance with a panel per variance ratio). A, Bias in estimate of a null treatment effect. B, Intra‐cluster correlation for group therapy where the data generating ICC, *ρ =* 0.05. C, Empirical coverage of a 95% C.I

In Figure 6A, we see that there was substantial bias for estimates of the treatment effect using the random effects instrumental variable regression method (RE‐IV). This bias increased dramatically with increased cluster size, but less so with increased intra‐cluster correlation (see appendix figure A8). Instrumental variable methods use two regression models. In this setting, the dependent variable of the random effects instrument model is equal to 1 in clusters and is zero otherwise so that all the variation of the instrument is between clusters implying an intra‐class correlation of 1. As a consequence, the instrument model gives much greater weight to *never‐takers* in intervention arm in the treatment effect model explaining this bias. This also explains why doubling cluster size had a much greater adverse effect than doubling the intra‐cluster correlation coefficient in figure A8 as it is the trial design rather than the ICC of the outcome measure that affects the instrument model. We surmize that this implementation of an IV regression model with a random effect in the instrument model is unsuitable for this design.

The mixture models that failed to account for any difference between the variance of the compliers and noncompliers in the group treatment arm (MM and RM‐MM) also showed bias where *λ ≠* 1. The magnitude of the bias was almost identical for methods with or without a random effect suggesting that the bias was associated with heteroscedasticity between the *compliers* and *never‐takers* rather than the effect of clustering. This was confirmed by appendix figure A8 as the bias associated with MM and RE‐MM did not change with increased cluster size and/or ICC. Figure A11(i) compares 70% and 80% compliance for all methods except RE‐IV. Noting the scale change compared to Figure 6A all methods performed well, although there is slight evidence that reduced compliance increased the bias of RE‐MM.

#### 4.5.2 Intra‐cluster correlation

In contrast to other causal methods, estimates of the intra‐cluster correlation for RE‐IV had a “quadratic” shape and a substantial bias, particularly where *λ =* 2/3 (see Figure 6B). The bias of RE‐IV increased with increased cluster size and/or ICC (appendix figure A9). In contrast, the three other methods were not affected by the mean of never‐takers, but the random effects mixture model (RE‐MM) estimates were biased where *λ* ≠ 1. The estimates for the RE‐MM that allows for heteroscedasticity (RE‐MM(Het)) and the Bloom CACE (RE‐BLM) were both close to the data generating value. Figure A11(ii) compares 70% and 80% compliance where *λ =* 1 for all methods except RE‐IV. Compliance did not appear to affect estimates of the ICC.

#### 4.5.3 Coverage

Figure 6C illustrates coverage for causal methods. The random effects instrumental variables model was excluded due to its substantial bias for the treatment effect and the ICC that resulted in dramatically reduced coverage. Where *λ* = 1, all other methods had coverage close to 95% with those methods that take account of clustering either by inclusion of a random effect or the use of robust standard errors tending to be better. Where *λ* ≠ 1, coverage was reduced for those methods with treatment effect bias. Results for larger clusters size and/or ICC are given in appendix figure A10. As cluster size and/or the ICC increased the coverage of some methods deteriorated including all those that did not account for clustering. The instrumental variable method with robust standard errors (IV(Rob)), the random effects based Bloom estimator (RE‐BLM), the heteroscedastic mixture model with robust standard errors MM(Het‐Rob), and the heteroscedastic random effects model (RE‐MM(Het)) all maintained good coverage. Figure A11(iii) compares 70% and 80% compliance where *λ =* 1 for all methods except RE‐IV from which it can be seen that compliance did not appear to affect coverage.

## 5 EXAMPLE: LOWER BACK PAIN TRIAL

Our motivating example was a randomized trial testing a group therapy for the treatment of lower back pain in which only actual group was recorded. Table 2 gives the estimates of the treatment effect for a visual analog (VAS) estimate of pain at 9 months for OLS and those methods of analysis using actual group. All data used for these analyses are listed in Appendix 4 in Supporting Information. VAS scores were square root transformed to remove skewness, so estimates are not comparable with the published report^11^, where the outcome data were not transformed. Data were available for 203 of 234 randomized subjects. All analyses used 203 subjects. Of these 105 patients had been randomized to group treatment of whom 71 adhered to the intervention, so the compliance rate was 68% (71/105) among patients with outcome data. Group therapy was delivered by 17 groups. The mean actual group size was 4.18 (71/17), which is similar to one of the cluster sizes in the simulated study.

**TABLE 2:** Lower back pain trial outcome measure—square root transformed visual analog scale (VAS) pain at 9 months adjusted for baseline VAS pain (for all analyses n = 203)

	Method	Treat. Effect	SE	z/t	p	rho
Intention to treat						
Linear model	OLS	−0.428	0.366	−1.17	0.244	
Linear model with robust standard errors	OLS (Rob‐Act)	−0.428	0.386	−1.11	0.270	
GEE model with exchangeable correlation matrix & robust standard errors	GEE(Act)	−0.370	0.380	−0.97	0.330	0.100
Random intercept model	RI(Act)	−0.340	0.394	−0.86	0.388	0.155
Random effects model with between arm heteroscedasticity	RE(Act)	−0.306	0.383	−0.80	0.424	0.134
Random effects model with heteroscedasticity between arms and within intervention arms	RE(Act‐Het)	−0.337	0.386	−0.87	0.383	0.150
Causal Models						
Bloom CACE estimator with random effects	RE‐BLM	−0.690	0.563	−1.23	0.220	0.119
Instrumental variables regression with or without robust standard errors	IV	−0.645	0.546	−1.18	0.239	
	IV(Rob)	−0.645	0.573	−1.13	0.262	
Instrumental variables regression with a random effect	RE‐IV	−0.587	0.703	−0.83	0.404	0.181
Mixture model without or with robust standard errors	MM	−0.563	0.549	−1.020	0.306	
	MM(Rob)	−0.563	0.628	−0.900	0.370	
Mixture model with between arm heteroscedasticity for compliers without or with robust standard errors	MM(Het)	−0.554	0.537	−1.030	0.302	
	MM(Het‐Rob)	−0.554	0.587	−0.940	0.345	
Mixture model with a random effect without or with heteroscedasticity for compliers	RE‐MM	−0.593	0.581	−1.020	0.307	0.072
	RE‐MM(Het)	−0.589	0.568	−1.040	0.300	0.120

From the simulation work, we have seen that the Bloom CACE estimator with random effects (RE‐BLM) and the mixture model with random effect with heteroscedasticity (RE‐MM(Het)) gave good estimates of the ICC. For these data, the estimates of the ICC were 0.119 and 0.120, respectively, which were smaller than the estimates of the ICC for the RI(Act), RE(Act), RE(Act‐Het)), but larger than the GEE estimate. The ICC estimate for the instrumental variables method with random effects (RE‐IV) was 0.181, which was larger than other methods consistent with the simulation results in Section 4.5.2.

Considering the treatment effect, those methods that adjust for clustering (GEE(Act), RI(Act), RE(Act), RE(Act‐Het)) were reduced in magnitude compared to the ITT methods that weight subjects equally (OLS, OLS(Rob‐Act)). GEE had the smallest reduction, which can be explained by its smaller ICC estimate. From the parameter estimates of the RE‐MM(Het) model, the difference between *never‐takers* and *compliers* (*γ*‐*τ*) is 1.32. This can be substituted into expression (11) to obtain a prediction of the treatment effect bias of the RE(Act) model of 0.097. This was smaller than the observed difference between the RE(Act) model and the OLS estimate of 0.122 (see Table 2), which is consistent with the results of the simulation study. From the parameter estimates of the RE(Act‐Het) model the variance ratio (*λ*) is 1.02. Substitution into expression (13) gives a prediction of the bias of the RE(Act‐Het) model of 0.125. This was rather larger than the observed difference between RE(Act‐Het) and OLS of 0.091, which is an illustration of the inconsistency of expression (13) also observed in the simulation work. The treatment effect estimates based on causal methods have greater magnitude as one would expect, but there are some differences with the Bloom CACE estimator being the largest and the mixture model (MM(Het) /MM(Het‐Rob)) being the smallest.

In this trial adjustment for clustering by using actual group has biased the ITT treatment effect estimates toward the null, but this may not always be true. If the outcome for *never‐takers* had been better than those receiving the group intervention, the bias could have been away from the null.

## 6 DISCUSSION

The work here was motivated by a trial of a group administered treatment for lower back pain. We suggest that it may also have relevance to trials of other forms of treatment related clustering such as talking and physical therapies and surgery where there is partial nesting and noncompliance, but cluster sizes may be larger or more variable than here.

Theoretical considerations suggest that some ITT methods used to account for clustering may be biased where an actual group analyses is used. It is suggested that this bias depends on the design effect, the rate of noncompliance, and the difference in outcome between *never‐takers* and *compliers* in the intervention arm (*γ*‐*τ*). This bias has been confirmed by a simulation study for random effects models and a GEE model with an exchangeable correlation matrix. What is more the magnitude of this bias was dependent on heteroscedasticity.

Random effects models and a GEE model using an exchangeable correlation matrix and actual group were unbiased in under certain conditions, but it is unlikely that one would know whether these applied prior to data analysis, so these methods are not recommended. In contrast, there was no evidence of treatment effect bias for OLS estimates and the use of robust standard errors based on actual group gave acceptable coverage.

We also considered ITT analyses based on the intended group. These models violate the notion that the clustering effect arises from group administration of treatment. Nevertheless, they showed no evidence of bias for the treatment effect. It has previously been shown that estimates of the clustering effect based on a random intercept models are biased in the presence of heteroscedasticity and use of a random effects model that allows for this is recommended.^7^, ^12^ This is supported by our simulation work. We would advise against the use of a random intercept model for an analyses based on the intended group. The random effects model (RE(Int)) gave slightly better coverage than the GEE model, and also the OLS model with robust standard errors. For a smaller cluster size and smaller intra‐cluster correlation coverage of the RE(Int) model was close to the desired value. There was a suggestion of reduced coverage for larger cluster size and intra‐cluster correlation, which might be a concern if groups were larger or the intra‐cluster correlation greater than the values considered here.

We also considered methods by which the causal treatment effect can be estimated. We saw that a standard implementation of an instrumental variable model with random effects performed very badly. A standard instrumental variables method with robust standard errors gave unbiased estimates of the treatment effect and good coverage, as did the Bloom CACE estimator that also gave an estimate of the clustering effect close to the desired value. Methods based on mixture models performed well provided they modelled heteroscedasticity and used robust standard errors (MM(Het‐Rob)) or a random effect (RE‐MM(Het), with the random effects model giving an unbiased estimate of the ICC.

Among ITT methods, our simulation work showed that OLS with robust standard errors using the actual group (OLS(Act)) gave the best coverage, but this was only a slight improvement over three methods using the intended clusters, a random coefficient model allowing for heteroscedasticity between arms (RE(Int)), OLS with robust standard errors(OLS(Int)), and GEE with an exchangeable correlation matrix (GEE(Int)). The small difference in coverage suggests that other considerations may be a more important for the choice of analysis method.

Where trials follow subjects over time, analyses based on longitudinal mixed models are recommended to address bias due to missing data. This suggests that there may be benefits of assigning therapy group membership at randomization so that intended group analyses can be applied using a mixed model. It should be noted, nevertheless, this work has made some assumptions that could favor an intended group analyses, which may not apply in practice. We have assumed that patients in the control arm did not receive the group intervention, so there are no *always‐takers*. Failure to take account of this by an intended group analysis could bias coverage as clustering in the control arm would not be included. We have also that patients in the intervention arm cannot switch between therapy groups. Where there is switching Walwyn and Roberts^13^ suggest used of multiple membership models.^28^ Roberts and Walwyn^29^ investigated the use of multiple membership models in the context of a partially nested design and showed that switching leads to underestimation of the clustering effect that adversely affects coverage. This suggests that trial protocols should prevent access to the group treatment by patients in the control arm and prevent switching between groups in the intervention arm. Where possible, compliance and the actual group in which a patient receives treatment should be recorded to confirm the robustness of an intended group analysis. This would also enable a causal model to be fitted as a sensitivity analysis to check the underlying data generation model. Such a sensitivity analysis could also be used where the ITT analysis has to be based on actual group, as worse outcome among *never‐takers* than *compliers* could allow one to infer that some ITT estimates are biased toward the null including GEE(Act), RI(Act), and RE(Act), although not RE(Act‐Het) as the direction of treatment effect bias was inconsistent. It should be noted that it may not be possible or appropriate to fit causal models as compliance may not be recorded or be recordable on a binary scale and the assumptions of such models may not be satisfied.^18^

Schweig and Pane^30^ investigated the effects of noncompliance in a partially nested design motivated by a randomized controlled trial of an educational intervention. In the intervention arm of their hypothetical study students attended summer training programs administered in classes that were nested in school, whereas students in the control arm were un‐clustered as they had independent summer experiences. Schweig and Pane's simulation work included *always‐takers* as well as *never‐takers*, as do we in Appendix A. They tested the effect of noncompliance on four methods of analysis. The first implements an intended group analysis (model A)^30^ similar to Equation (4). The second (model E)^30^ is similar to the random effects model defined by Equation (5). Their two other models (models F and G)^30^ include the variables for *never‐taker* or *always‐taker* used to generate data. These compliance variables are only partially observed in real data as the *never‐taker* variable is only observed in the intervention arm and the *always‐taker* variable only in the control, so models F and G cannot be fitted to trial data. Instead, it requires the use of mixture models where compliance data cannot be observed.

Schweig and Pane^30^ found that there was bias for their model E where they simulated very substantial heteroscedasticity, but this is only a partial explanation. A limitation of their simulation study is that they did not investigate the effect of differences between *complier*s, *never‐takers*, and *always‐takers*. In Appendix A, we derived an expression for the treatment effect bias where there are *complier*s, *never‐takers*, and *always‐takers*. Expression (A3) suggests that this bias is a function of two terms; the difference between *never‐takers* and *compliers* and the difference between *always‐takers* and *compliers*. Schweig and Pane's models F performed well as heteroscedasticity was modeled, which is consistent with our simulation work based on the mixture model, RE‐MM(Het). Schweig and Pane's models F appeared to be affected by heteroscedasticity as was the corresponding mixture model in our study, RE‐MM.

The ITT methods of analysis that we have considered here could be applied to the setting where there are *always‐takers* as well as *never‐takers*. Models using robust standard errors could be implemented with minimal adaption. Random effects and GEE models with an exchangeable correlation matrix based on actual group need to consider the implications of the bias suggested by expression (A3) in Appendix A. As mentioned above, inclusion of *always‐takers* may affect coverage of intended group analyses. Considering causal methods, the Bloom CACE method described by Equation (14) would require an additional indicator variable identifying the *always‐takers* in the control arm and an additional residual variance term for this sub‐group to account for heteroscedasticity. The mixture model methods would require a second mixture model in the intervention arm to separate the *always‐takers* from *compliers*.

In this study, we have seen that the weighting of data by the methods of analysis may induce bias where the outcome of subjects in clusters differs from those that are not. This can be viewed as an example of informative clustering, which introduces bias because the pattern of clustering differs between treatment arms. In some settings, the weighting of subjects by the design effect should not cause bias. For example, in cluster randomized trials, the method of allocation provides protection against informative cluster size as the characteristics of clusters should be balanced between trial arms. In observational data, informative cluster size and the intra‐cluster correlation are more problematic, as it could be confounded with the study effect of interest in ways that could cause bias.^31^, ^32^

The issues raised in this article are due in part to such trials not being fully randomized. In a tightly controlled designed experiment, subjects would be randomized to therapy group as well as intervention arm and then the analysis based on the experimental factors. The ability to do this would resolve some of the issues, but such designs are rarely feasible in a clinical setting.

We conclude by emphasizing the importance of considering the analysis implications of noncompliance at the design stage. While practical constraints of the clinical setting may determine the choice between an intended or actual group analyses, there could be benefits of recording both so that a primary ITT analysis could use the intended group, while a sensitivity analysis based on actual group might check the data generating mechanism if compliance. Finally, it should always be remembered that reducing the rate of noncompliance is desirable for internal validity and maintaining statistical power, whichever method is used.

## Supporting information


**Data S1:** Appendix 2

**Data S2:** Appendix 3

**Data S3:** Appendix 4

## Data Availability

In Section 5, there is the statement “All data used for these analyses are listed in Appendix 4 in Supporting information.”

## Endnote

1 The term *group* refers to *therapy group* not *intervention arm*.

## APPENDIX A TREATMENT EFFECT BIAS IN AN ACTUAL GROUP ANALYSIS OF PARTIAL NESTING DESIGN

We assume monotonicity so there are just three compliance classes, *compliers*, *never‐takers*, and *always‐takers*.^14^ Randomization means that the prevalence of each category is the same in each arm, say *π*_*c*_, *π*_*n*_, and *π*_*a*._ We also assume that randomization only influences the patient's outcome by the receipt of the group treatment, an assumption referred to as the *exclusion restriction*, so the mean outcome for *never‐takers* and *always‐takers* is independent of randomization.

Let us suppose that the mean outcome for the subjects in the control arm is *μ* and the causal effect of treatment is*τ* so the mean outcome for compliers in the intervention arm is *μ + τ*. Let us assume the mean outcome is *μ + γ*_*n*_ for *never‐takers* and *μ + γ*_*a*_ *+ τ* for *always‐takers*. Suppose *ρ* is the data generating ICC for group therapy with $\rho =\sigma_{u}^{2}/\left(\sigma_{u}^{2}+\sigma_{T}^{2}\right)$ as define by Equations (9). Assuming equal size therapy groups, say *m*, the design effect *D* = 1 + (*m* − 1)*ρ*.

Random effects models fitted using maximum likelihood estimation and GEE, down‐weight subjects in clusters by 1/*D* compared to nonnested subjects in the intervention arm. The mean outcome for each arm can be expressed as a weighted mean, from which we can obtain an approximation to the estimated ITT effect, say *δ*_*w*_.

(A1) $$\delta_{w}=\frac{\frac{\left(\mu +\tau \right)\pi_{c}}{D}+\left(\mu +\gamma_{n}\right)\pi_{n}+\frac{\left(\mu +\gamma_{a}+\tau \right)\pi_{a}}{D}}{\frac{\pi_{c}}{D}+\pi_{n}+\frac{\pi_{a}}{D}\;}-\frac{{\mu \pi }_{c}+\left(\mu +\gamma_{n}\right)\pi_{n}+\frac{\left(\mu +\gamma_{a}+\tau \right)\pi_{a}}{D}}{\pi_{c}+\pi_{n}+\frac{\pi_{a}}{D}\;}.$$

After some rearrangements, we obtain the expression

(A2) $$\delta_{w}=\pi_{c}\left(\frac{\tau D+\gamma_{n}\pi_{n}D\left(D-1\right)+\gamma_{a}\pi_{a}\left(D-1\right)}{\left(1-\pi_{n}+\pi_{n}D\right)\left(\pi_{a}+\left(1-\pi_{a}\right)D\right)}\right).$$

If subjects were weighted equally, the intentions‐to‐treat effect would be *δ*_*e*_ = *τπ*_*c*_. Bias of random effects models or GEE compared to equal weighting of subjects is therefore

(A3) $$\delta_{w}-\delta_{e}=\pi_{c}\left(\frac{\gamma_{n}\pi_{n}D\left(D-1\right)+\gamma_{a}\pi_{a}\left(D-1\right)+\tau D}{\left(1-\pi_{n}+\pi_{n}D\right)\left(\pi_{a}+\left(1-\pi_{a}\right)D\right)}-\tau \right).$$

Assuming *ρ* > 0, the denominator of (A2) and (A3) are positive as are *D* and *D*−1. The approximation *δ*_*w*_ is therefore a linear function increasing in *γ*_*n*_ and *γ*_*a*_.

In the design we are considering here there are no *always‐takers*, that is, *π*_*a*_ = 0. Replacing γ_*n*_ with *γ*,  *π*_*c*_ with *π* and substituting *π*_*n*_ = 1 − *π* gives Equation (10) in the main text

(A4) $$\delta_{w}=\pi_{c}\left(\frac{\tau +\gamma \left(1-\pi \right)\left(D-1\right)}{\pi +\left(1-\pi \right)D}\right).$$

Similarly

(A5) $$\delta_{w}-\delta_{e}=\left(\gamma -\tau \right)\left(\frac{\pi \left(1-\pi \right)\left(D-1\right)}{\pi +\left(1-\pi \right)D}\right)\;,$$

which is Equation (11) in the main text.

Equations (3), (5), and (6) estimate $\sigma_{u}^{2}$ about the mean or adjusted mean of the intervention arm. Unless *γ* = *τ*, the estimate of $\sigma_{u}^{2}$ based on Equations (3), (5), and (6) could be inflated by the square of the difference between the mean of compliers in the intervention arm and the mean of the intervention arm. From the first term on the right hand side of Equation (A3), the mean of the intervention arm is given by

$$\mu +\left(\frac{\pi \tau +\gamma \left(1-\pi \right)D}{\pi +\left(1-\pi \right)D}\right).$$

The data generating mean of the compliers in the intervention arm is *μ* + *τ*. Hence the difference between the intervention arm mean and the data generating mean of compliers in the intervention arm can be approximated by:

$$\mu +\left(\frac{\pi \tau +\gamma \left(1-\pi \right)D}{\pi +\left(1-\pi \right)D}\right)-\left(\mu +\tau \right),$$

which simplifies to

$$\left(\frac{\left(\gamma -\tau \right)\left(1-\pi \right)D}{\pi +\left(1-\pi \right)D}\right).$$

If $\sigma_{u}^{2}$ is inflated by this difference squared, the estimated the estimate of *ρ* for Equations (3) and (5) will be

(A6) $$\left(\frac{\rho +{\left(\frac{\left(\gamma -\tau \right)\left(1-\pi \right)D}{\left(\pi +\left(1-\pi \right)D\right)}\right)}^{2}}{1+{\left(\frac{\left(\gamma -\tau \right)\left(1-\pi \right)D}{\left(\pi +\left(1-\pi \right)D\right)}\right)}^{2}}\right)\;,$$

which is expression (12) in the main text. For Equation (6), we can replace D by *λ* *D*, where $\lambda =\left(\sigma_{e1}^{2}+\sigma_{u}^{2}\right)/\sigma_{e2}^{2}$.

This is a crude approximation as biased estimation $\sigma_{u}^{2}$ would affect estimates of other variance terms. It does nevertheless suggest that that any estimation bias of $\sigma_{u}^{2}$ could be a quadratic in *γ*.
