# Supplementary material for: The implications of noncompliance for randomized trials with partial nesting due to group treatment
Source: Stat Med. 2020 Oct 28;40(2):349–68. doi: 10.1002/sim.8778 (PMC7821326; doi:10.1002/sim.8778)
Supplement: Supplementary file 1 — Data S1: Appendix 2 [file SIM-40-349-s001.docx]

**Appendix 2: Supplementary Figures**

|  | **Figure A1** Actual Cluster: Bias for Null Treatment Effect (200 subjects per arm and 80% compliance and a panel per variance ratio). | |
| --- | --- | --- |
|  | Group size=5 | Group size=10 |
| ICC= 0.05 |  |  |
| ICC =0.1 |  |  |

|  | **Figure A2** Actual Cluster: Comparison with Predicted Treatment Effects (200 subjects per arm and 80% compliance and a panel per variance ratio). | |
| --- | --- | --- |
|  | Group size=5 | Group size=10 |
| ICC= 0.05 |  |  |
| ICC =0.1 |  |  |

|  | **Figure A3** Actual Cluster: Intra-cluster correlation (200 subjects per arm and 80% compliance and a panel per variance ratio). | |
| --- | --- | --- |
|  | Group size=5 | Group size=10 |
| ICC= 0.05 |  |  |
| ICC =0.1 |  |  |

|  | **Figure A4** Actual Cluster: 95% C.I. Coverage (200 subjects per arm and 80% compliance and a panel per variance ratio). | |
| --- | --- | --- |
|  | Group size=5 | Group size=10 |
| ICC= 0.05 |  |  |
| ICC =0.1 |  |  |
|  | Scale change for group size=10 and ICC =0.1. | |

|  | **Figure A5** Intended Cluster: Bias for Null Treatment Effect (200 subjects per arm and 80% compliance and a panel per variance ratio). | |
| --- | --- | --- |
|  | Group size=5 | Group size=10 |
| ICC= 0.05 |  |  |
| ICC =0.1 |  |  |

|  | **Figure A6** Intended Cluster: Intra-cluster correlation (200 subjects per arm and 80% compliance and a panel per variance ratio). | |
| --- | --- | --- |
|  | Group size=5 | Group size=10 |
| ICC= 0.05 |  |  |
| ICC =0.1 |  |  |
|  |  |  |

|  | **Figure A7** Intended Cluster: 95% C.I. Coverage (200 subjects per arm and 80% compliance and a panel per variance ratio). | |
| --- | --- | --- |
|  | Group size=5 | Group size=10 |
| ICC= 0.05 |  |  |
| ICC =0.1 |  |  |

|  | **Figure A8** Causal Models with Actual Cluster: Null Treatment Effect (200 subjects per arm and 80% compliance with a panel per variance ratio). | |
| --- | --- | --- |
|  | Group size=5 | Group size=10 |
| ICC= 0.05 |  |  |
| ICC =0.1 |  |  |

|  | **Figure A9** Causal Models with Actual Cluster: Intra-cluster correlation (200 subjects per arm and 80% compliance with a panel per variance ratio). | |
| --- | --- | --- |
|  | Group size=5 | Group size=10 |
| ICC= 0.05 |  |  |
| ICC =0.1 |  |  |

|  | **Figure A10** Causal Models with Actual Cluster: 95% C.I. Coverage (200 subjects per arm and 80% compliance with a panel per variance ratio). | |
| --- | --- | --- |
|  | Group size=5 | Group size=10 |
| ICC= 0.05 |  |  |
| ICC =0.1 |  |  |
|  | Scale change for group size=10 and ICC =0.1. | |

| **Figure A11** Causal Models with Actual Cluster: Comparison of 70% and 80% compliance (200 subjects per arm, Group size 5, ICC 0.05, Equal Variance) | |
| --- | --- |
| 1. Bias in estimate of a null treatment effect |  |
| 1. Intra-cluster correlation for group therapy where the data generating ICC,  |  |
| 1. Empirical Coverage of a 95% C.I. |  |
