# Supplementary material for: The implications of noncompliance for randomized trials with partial nesting due to group treatment
Source: Stat Med. 2020 Oct 28;40(2):349–68. doi: 10.1002/sim.8778 (PMC7821326; doi:10.1002/sim.8778)
Supplement: Supplementary file 2 — Data S2: Appendix 3 [file SIM-40-349-s002.docx]

**Appendix 3: Stata Code For Data Generation and Analysis Method**

*! version 1.2 Oct 11th 2019

program define pn_compliance, rclass

syntax [,m(integer 1) k(integer 1) n(integer 1) mean_c(real 1) mean_nc(real 0) treat_eff(real 0) /*

*/ sd_c(real 1) sd_nc(real 1) landa(real 1) rho(real 0) p_comply(real 1) mm xtmm mm2 xtmm2 intended capt(string)]

drop _all

*

* generation of partially nested data with non-compliance

*

set obs 2

gen arm= _n -1

expand `k' if arm==1

gen u=invnorm(uniform()) if arm==1

gen cluster_int= _n -1

gen cluster_act=cluster_int

gen m=`m'

expand m if arm==1

expand `n' if arm==0

sort arm

replace cluster_int= `k'+_n if arm==0

gen complier= (uniform()<`p_comply' )

gen treatrec=complier*arm

gen treatnotrec=(1-complier)*arm

sort treatrec

replace cluster_act= `k'+_n if treatrec==0

sum treatrec if arm==1

local est_p_comply = r(mean)

return scalar est_p_comply = r(mean)

* compliers in the control

gen y=`mean_c'+`sd_c'*invnorm(uniform()) if complier==1 & arm==0

* noncompliers

replace y=`mean_nc'+`sd_nc'*invnorm(uniform()) if complier==0

*compliers in the intervention

replace y=`mean_c' + `treat_eff'+ sqrt(`landa')*`sd_c'*(sqrt(1-`rho')*invnorm(uniform()) + sqrt(`rho')*u) if treatrec==1

gen procedure =0

*

* Summary statistics

*

table complier arm,c(mean y sd y n y mean u sd u)

loneway y cluster_int if arm==1

return scalar rho_int=r(rho)

loneway y cluster_act if arm==1

return scalar rho_act_arm=r(rho)

loneway y cluster_act if treatrec==1

return scalar rho_act_receive=r(rho)

preserve

collapse (mean) treatrec (count) y, by( cluster_act )

tab y

sum y if treatrec==1

local mmean_act=r(mean)

return scalar mmean_act=r(mean)

local mvar_act=r(Var)

return scalar mvar_act=r(Var)

restore

*****************************************************

*

* Statistical Analysis

*

*****************************************************

*

* OLS

*

`capt' xi:reg y arm

local b_itt =_b[arm]

return scalar b_itt =_b[arm]

local se_itt =_se[arm]

return scalar se_itt =_se[arm]

*****************************************************

*

* Analyses using Actual group

*

*****************************************************

*

* OLS(Rob-Act)

*

`capt' xi:reg y arm , cluster(cluster_act)

local b_itt_act =_b[arm]

return scalar b_itt_act=_b[arm]

local se_itt_act =_se[arm]

return scalar se_itt_act =_se[arm]

*

*Random Intercept Model RI(Act)

*

`capt' xi:xtreg y arm ,i(cluster_act) mle

local b_xtreg_act =_b[arm]

return scalar b_xtreg_act =_b[arm]

local se_xtreg_act =_se[arm]

return scalar se_xtreg_act =_se[arm]

local rho_xtreg_act =e(rho)

return scalar rho_xtreg_act =e(rho)

pause

*

*Random Effects Model RE(Act)

*

`capt' xtmixed y arm ||cluster_act:treatrec , var nocons iter(25) technique(nr) matsqrt residual(inde,by(arm)) reml

local b_xtmixed_rc_act =_b[arm]

return scalar b_xtmixed_rc_act =_b[arm]

local se_xtmixed_rc_act =_se[arm]

return scalar se_xtmixed_rc_act =_se[arm]

local vare1_xtmixed_rc_act=exp(_b[lnsig_e:_cons])^2

return scalar vare1_xtmixed_rc_act=`vare1_xtmixed_rc_act'

local vare2_xtmixed_rc_act=exp(_b[lnsig_e:_cons]+_b[r_lns2ose:_cons])^2

return scalar vare2_xtmixed_rc_act=`vare2_xtmixed_rc_act'

local varu_xtmixed_rc_act=exp(_b[lns1_1_1:_cons])^2

return scalar varu_xtmixed_rc_act=`varu_xtmixed_rc_act'

local rho_xtmixed_rc_act= `varu_xtmixed_rc_act'/(`vare2_xtmixed_rc_act'+ `varu_xtmixed_rc_act')

return scalar rho_xtmixed_rc_act=`rho_xtmixed_rc_act'

*

*Random Effects Model RE(Act-Het)

*

gen group=arm+treatrec

`capt' xtmixed y arm ||cluster_act:treatrec , var nocons iter(25) technique(nr) matsqrt residual(inde,by(group)) reml

local b_xtmixed_act =_b[arm]

return scalar b_xtmixed_act =_b[arm]

local se_xtmixed_act =_se[arm]

return scalar se_xtmixed_act =_se[arm]

local vare1_xtmixed_act=exp(_b[lnsig_e:_cons])^2

return scalar vare1_xtmixed_act=`vare1_xtmixed_act'

local vare2_xtmixed_act=exp(_b[lnsig_e:_cons]+_b[r_lns2ose:_cons])^2

return scalar vare2_xtmixed_act=`vare2_xtmixed_act'

local vare3_xtmixed_act=exp(_b[lnsig_e:_cons]+_b[r_lns3ose:_cons])^2

return scalar vare3_xtmixed_act=`vare3_xtmixed_act'

local varu_xtmixed_act=exp(_b[lns1_1_1:_cons])^2

return scalar varu_xtmixed_act=`varu_xtmixed_act'

local rho_xtmixed_act= `varu_xtmixed_act'/(`vare3_xtmixed_act'+ `varu_xtmixed_act')

return scalar rho_xtmixed_act=`rho_xtmixed_act'

*

* GEE Model GEE(Act)

*

`capt' xi:xtgee y arm ,i(cluster_act) robust

local b_xtgee_act =_b[arm]

return scalar b_xtgee_act =_b[arm]

local se_xtgee_act =_se[arm]

return scalar se_xtgee_act =_se[arm]

matrix X =e(R)

local rho_xtgee_act = X[1,2]

return scalar rho_xtgee_act =`rho_xtgee_act'

`capt' xi:xtgee y arm ,i(cluster_act) robust corr(ind)

local b_xtgee_ind_act =_b[arm]

return scalar b_xtgee_ind_act =_b[arm]

local se_xtgee_ind_act =_se[arm]

return scalar se_xtgee_ind_act =_se[arm]

matrix X =e(R)

local rho_xtgee_ind_act = X[1,2]

return scalar rho_xtgee_ind_act =`rho_xtgee_ind_act'

*****************************************************

*

* Analyses using intended cluster

*

*****************************************************

if "`intended'"!="" {

*

* OLS(Rob-Int)

*

`capt' xi:reg y arm , cluster(cluster_int)

local b_itt_int =_b[arm]

return scalar b_itt_int =_b[arm]

local se_itt_int =_se[arm]

return scalar se_itt_int =_se[arm]

*

* Random Intercept Model RI(Int)

*

`capt' xi:xtreg y arm ,i(cluster_int) mle

local b_xtreg_int =_b[arm]

return scalar b_xtreg_int =_b[arm]

local se_xtreg_int =_se[arm]

return scalar se_xtreg_int =_se[arm]

local rho_xtreg_int =e(rho)

return scalar rho_xtreg_int =e(rho)

pause

*

* Random Effects Model RE(Int)

*

`capt' xtmixed y arm ||cluster_int:arm , var nocons iter(25) technique(nr) matsqrt residual(inde,by(arm)) reml

local b_xtmixed_int =_b[arm]

return scalar b_xtmixed_int =_b[arm]

local se_xtmixed_int =_se[arm]

return scalar se_xtmixed_int =_se[arm]

local vare1_xtmixed_int=exp(_b[lnsig_e:_cons])^2

return scalar vare1_xtmixed_int=`vare1_xtmixed_int'

local vare2_xtmixed_int=exp(_b[lnsig_e:_cons]+_b[r_lns2ose:_cons])^2

return scalar vare2_xtmixed_int=`vare2_xtmixed_int'

local varu_xtmixed_int=exp(_b[lns1_1_1:_cons])^2

return scalar varu_xtmixed_int=`varu_xtmixed_int'

local rho_xtmixed_int= `varu_xtmixed_int'/(`vare2_xtmixed_int'+ `varu_xtmixed_int')

return scalar rho_xtmixed_int=`rho_xtmixed_int'

*

* GEE Intended Cluster GEE(Int)

*

`capt' xi:xtgee y arm ,i(cluster_int) robust

local b_xtgee_int =_b[arm]

return scalar b_xtgee_int =_b[arm]

local se_xtgee_int =_se[arm]

return scalar se_xtgee_int =_se[arm]

matrix X =e(R)

local rho_xtgee_int = X[1,2]

return scalar rho_xtgee_int = X[1,2]

`capt' xi:xtgee y arm ,i(cluster_int) robust corr(ind)

local b_xtgee_ind_int =_b[arm]

return scalar b_xtgee_ind_int =_b[arm]

local se_xtgee_ind_int =_se[arm]

return scalar se_xtgee_ind_int =_se[arm]

matrix X =e(R)

local rho_xtgee_ind_int = X[1,2]

return scalar rho_xtgee_ind_int = X[1,2]

*****************************************************

*

* Causal Methods

*

*****************************************************

*

* IV

*

`capt' xi:ivreg y ( treatrec = arm ) ,first

local b_iv = _b[treatrec]

return scalar b_iv = _b[treatrec]

local se_iv = _se[treatrec]

return scalar se_iv = _se[treatrec]

*

* IV(Rob)

*

`capt' xi:ivreg y ( treatrec = arm ) , cluster(cluster_act) first

local b_iv_act = _b[treatrec]

return scalar b_iv_act =_b[treatrec]

local se_iv_act = _se[treatrec]

return scalar se_iv_act= _se[treatrec]

*

* Random Effects IV with Actual cluster RE-IV

*

`capt' xi:xtivreg y ( treatrec = arm ) ,i(cluster_act) first

local b_xtiv_act = _b[treatrec]

return scalar b_xtiv_act = _b[treatrec]

local se_xtiv_act = _se[treatrec]

return scalar se_xtiv_act = _se[treatrec]

local mc_xtiv_act = _b[_cons]

return scalar mc_xtiv_act = _b[_cons]

local rho_xtiv_act = e(rho)

return scalar rho_xtiv_act = e(rho)

*

* Instrumental variable with intended cluster

*

`capt' xi:ivreg y ( treatrec = arm ) , cluster(cluster_int) first

local b_iv_int = _b[treatrec]

return scalar b_iv_int=_b[treatrec]

local se_iv_int = _se[treatrec]

return scalar se_iv_int= _se[treatrec]

*

* Random Effects IV with Intended cluster

*

xi:xtivreg y ( treatrec = arm ) ,i(cluster_int) first

local b_xtiv_int = _b[treatrec]

return scalar b_xtiv_int = _b[treatrec]

local se_xtiv_int = _se[treatrec]

return scalar se_xtiv_int = _se[treatrec]

local mc_xtiv_int = _b[_cons]

return scalar mc_xtiv_int = _b[_cons]

local rho_xtiv_int = e(rho)

return scalar rho_xtiv_int = e(rho)

}

*

*Random Effects Bloom CACE Model RE-BLM

*

`capt' xtmixed y treatrec treatnotrec ||cluster_act:treatrec , var nocons iter(25) technique(nr) matsqrt residual(inde,by(group)) reml

local b_xtmixed_treatrec =_b[treatrec]

return scalar b_xtmixed_treatrec =_b[treatrec]

local se_xtmixed_treatrec =_se[treatrec]

return scalar se_xtmixed_treatrec =_se[treatrec]

local b_xtmixed_treatnotrec =_b[treatnotrec]

return scalar b_xtmixed_treatnotrec =_b[treatnotrec]

local se_xtmixed_treatnotrec =_se[treatnotrec]

return scalar se_xtmixed_treatnotrec =_se[treatnotrec]

lincom `est_p_comply'*_b[treatrec]+(1-`est_p_comply')*_b[treatnotrec]

local b_xtmixed_wt1 =r(estimate)

return scalar b_xtmixed_wt1 =r(estimate)

local se_xtmixed_wt1= r(se)

return scalar se_xtmixed_wt1 = r(se)

lincom (`est_p_comply'*_b[treatrec]+(1-`est_p_comply')*_b[treatnotrec])/`est_p_comply'

local b_xtmixed_wt2 =r(estimate)

return scalar b_xtmixed_wt2 =r(estimate)

local se_xtmixed_wt2= r(se)

return scalar se_xtmixed_wt2 = r(se)

local vare1_xtmixed_wt=exp(_b[lnsig_e:_cons])^2

return scalar vare1_xtmixed_wt=`vare1_xtmixed_wt'

local vare2_xtmixed_wt=exp(_b[lnsig_e:_cons]+_b[r_lns2ose:_cons])^2

return scalar vare2_xtmixed_wt=`vare2_xtmixed_wt'

local vare3_xtmixed_wt=exp(_b[lnsig_e:_cons]+_b[r_lns3ose:_cons])^2

return scalar vare3_xtmixed_wt=`vare3_xtmixed_wt'

local varu_xtmixed_wt=exp(_b[lns1_1_1:_cons])^2

return scalar varu_xtmixed_wt=`varu_xtmixed_wt'

local rho_xtmixed_wt= `varu_xtmixed_wt'/(`vare3_xtmixed_wt'+ `varu_xtmixed_wt')

return scalar rho_xtmixed_wt=`rho_xtmixed_wt'

*****************************************************

*

* Mixture Models

*

*****************************************************

*

* Mixture model MM

*

if "`mm'"!=""|"`xtmm'"!=""{

ml model lf mm (y=arm) (arm=) (complier=) () ()

ml init eq1:_cons=0 eq1:arm=0 eq2:_cons=0 eq3:_cons=1 eq4:_cons=1 eq5:_cons=0

`capt' ml maximize,difficult tolerance(1e-4) nrtolerance(1e-5) iter(30)

local b_mm = _b[eq1:arm]

return scalar b_mm = _b[eq1:arm]

local se_mm = _se[eq1:arm]

return scalar se_mm = _se[eq1:arm]

local prob_mm = exp(_b[eq5:_cons])/(1+exp(_b[eq5:_cons]))

return scalar prob_mm = `prob_mm'

local mc_mm = _b[eq1:_cons]

return scalar mc_mm = _b[eq1:_cons]

local mn_mm = _b[eq2:_cons]

return scalar mn_mm = _b[eq2:_cons]

local sdc_mm =_b[eq3:_cons]

return scalar sdc_mm =_b[eq3:_cons]

local sdn_mm =_b[eq4:_cons]

return scalar sdn_mm =_b[eq4:_cons]

return scalar niter_mm=e(ic)

* local mm_fail=_rc

* return scalar mm_fail=_rc

*

* mixture model CACE robust MM(Rob)

*

ml model lf mm (y=arm) (arm=) (complier=) () () ,cluster(cluster_act)

ml init eq1:_cons=0 eq1:arm=0 eq2:_cons=0 eq3:_cons=1 eq4:_cons=1 eq5:_cons=0

`capt' ml maximize,difficult tolerance(1e-4) nrtolerance(1e-5) iter(30)

local b_mmr = _b[eq1:arm]

return scalar b_mmr = _b[eq1:arm]

local se_mmr = _se[eq1:arm]

return scalar se_mmr = _se[eq1:arm]

local prob_mmr = exp(_b[eq5:_cons])/(1+exp(_b[eq5:_cons]))

return scalar prob_mmr = `prob_mmr'

local mc_mmr = _b[eq1:_cons]

return scalar mc_mmr = _b[eq1:_cons]

local mn_mmr = _b[eq2:_cons]

return scalar mn_mmr = _b[eq2:_cons]

local sdc_mmr =_b[eq3:_cons]

return scalar sdc_mmr =_b[eq3:_cons]

local sdn_mmr =_b[eq4:_cons]

return scalar sdn_mmr =_b[eq4:_cons]

return scalar niter_mmr=e(ic)

if "`xtmm'"!=""{

*

* Random effects mixture model RE-MM

*

global MY_panel cluster_act

local lsdc_mm=ln(`sdc_mm')

local lsdn_mm=ln(`sdn_mm')

local lor_mm = _b[eq5:_cons]

global MY_panel cluster_act

ml model d1 re_mm (y=arm) (arm=) (complier=) () () ()

ml init eq1:arm=`b_mm' eq1:_cons=`mc_mm' eq2:_cons=`mn_mm' eq3:_cons=0 eq4:_cons=`lsdc_mm' eq5:_cons=`lsdn_mm' eq6:_cons=`lor_mm'

`capt' ml maximize, tolerance(1e-4) nrtolerance(1e-5) iter(30)

local b_xtmm = _b[eq1:arm]

return scalar b_xtmm = _b[eq1:arm]

local se_xtmm = _se[eq1:arm]

return scalar se_xtmm = _se[eq1:arm]

local prob_xtmm = exp(_b[eq6:_cons])/(1+exp(_b[eq6:_cons]))

return scalar prob_xtmm =`prob_xtmm'

local rho_xtmm=exp(_b[eq3:_cons])^2/(exp(_b[eq3:_cons])^2+exp(_b[eq4:_cons])^2)

return scalar rho_xtmm=`rho_xtmm'

local mc_xtmm=_b[eq1:_cons]

return scalar mc_xtmm=`mc_xtmm'

local mn_xtmm = _b[eq2:_cons]

return scalar mn_xtmm = `mn_xtmm'

local sdu_xtmm =exp(_b[eq3:_cons])

return scalar sdu_xtmm=`sdu_xtmm'

local sdc_xtmm =exp(_b[eq4:_cons])

return scalar sdc_xtmm=`sdc_xtmm'

local sdn_xtmm =exp(_b[eq5:_cons])

return scalar sdn_xtmm=`sdn_xtmm'

return scalar niter_xtmm=e(ic)

}

}

if "`mm2'"!=""| "`xtmm2'"!="" {

*

* mixture model allowing for heteroscedasticity MM(Het)

*

global MY_panel cluster_act

ml model lf mm_het (y=arm) (arm=) (complier=) () () ()

ml init eq1:_cons=0 eq1:arm=0 eq2:_cons=0 eq3:_cons=1 eq4:_cons=1 eq5:_cons=1 eq6:_cons=0

`capt' ml maximize,difficult tolerance(1e-4) nrtolerance(1e-5) iter(30)

local b_mm2 = _b[eq1:arm]

return scalar b_mm2 = _b[eq1:arm]

local se_mm2 = _se[eq1:arm]

return scalar se_mm2 = _se[eq1:arm]

local prob_mm2 = exp(_b[eq5:_cons])/(1+exp(_b[eq5:_cons]))

return scalar prob_mm2 = `prob_mm2'

local mc_mm2 = _b[eq1:_cons]

return scalar mc_mm2 = _b[eq1:_cons]

local mn_mm2 = _b[eq2:_cons]

return scalar mn_mm2 = _b[eq2:_cons]

local sdc_mm2 =_b[eq3:_cons]

return scalar sdc_mm2 =_b[eq3:_cons]

local sdn_mm2 =_b[eq4:_cons]

return scalar sdn_mm2 =_b[eq4:_cons]

local sdt_mm2 = _b[eq5:_cons]

return scalar sdt_mm2 = _b[eq5:_cons]

local landa_mm2=(`sdt_mm2'/`sdc_mm2')^2

return scalar landa_mm2= `landa_mm2'

return scalar niter_mm2=e(ic)

*

* mixture model allowing for heteroscedasticity with Robust SE MM(Het)

*

ml model lf mm_het (y=arm) (arm=) (complier=) () () () ,cluster(cluster_act)

ml init eq1:_cons=0 eq1:arm=0 eq2:_cons=0 eq3:_cons=1 eq4:_cons=1 eq5:_cons=1 eq6:_cons=0

`capt' ml maximize,difficult tolerance(1e-4) nrtolerance(1e-5) iter(30)

local b_mm2r = _b[eq1:arm]

return scalar b_mm2r = _b[eq1:arm]

local se_mm2r = _se[eq1:arm]

return scalar se_mm2r = _se[eq1:arm]

local prob_mm2r = exp(_b[eq5:_cons])/(1+exp(_b[eq5:_cons]))

return scalar prob_mm2r = `prob_mm2r'

local mc_mm2r = _b[eq1:_cons]

return scalar mc_mm2r = _b[eq1:_cons]

local mn_mm2r = _b[eq2:_cons]

return scalar mn_mm2r = _b[eq2:_cons]

local sdc_mm2r =_b[eq3:_cons]

return scalar sdc_mm2r =_b[eq3:_cons]

local sdn_mm2r =_b[eq4:_cons]

return scalar sdn_mm2r =_b[eq4:_cons]

local sdt_mm2r = _b[eq5:_cons]

return scalar sdt_mm2r = _b[eq5:_cons]

local landa_mm2r=(`sdt_mm2r'/`sdc_mm2r')^2

return scalar landa_mm2r= `landa_mm2r'

return scalar niter_mm2r=e(ic)

*

* Random effects mixture model allowing for Heteroscedasticity RE-MM(Het)

*

if "`xtmm2'"!=""{

gen xtmm2=1

local lsdc_mm2=ln(`sdc_mm2')

local lsdn_mm2=ln(`sdn_mm2')

local lsdt_mm2=ln(`sdt_mm2')

local lor_mm2 = _b[eq6:_cons]

local lsdu_xtmm=ln(`sdu_xtmm')

global MY_panel cluster_act

ml model d1 re_mm_het (y=arm) (arm=) (complier=) () () () ()

ml init eq1:arm=`b_mm2' eq1:_cons=`mc_mm2' eq2:_cons=`mn_mm2' eq3:_cons=`lsdu_xtmm' eq4:_cons=`lsdc_mm2' eq5:_cons=`lsdn_mm2' eq6:_cons=`lsdt_mm2' eq7:_cons=`lor_mm2'

`capt' ml maximize,tolerance(1e-4) nrtolerance(1e-5) iter(30)

local b_xtmm2 = _b[eq1:arm]

return scalar b_xtmm2 = _b[eq1:arm]

local se_xtmm2 = _se[eq1:arm]

return scalar se_xtmm2 = _se[eq1:arm]

local prob_xtmm2 = exp(_b[eq7:_cons])/(1+exp(_b[eq7:_cons]))

return scalar prob_xtmm2 =`prob_xtmm2'

local rho_xtmm2=exp(_b[eq3:_cons])^2/(exp(_b[eq3:_cons])^2+exp(_b[eq6:_cons])^2)

return scalar rho_xtmm2=`rho_xtmm2'

local mc_xtmm2=_b[eq1:_cons]

return scalar mc_xtmm2=`mc_xtmm2'

local mn_xtmm2 = _b[eq2:_cons]

return scalar mn_xtmm2 = `mn_xtmm2'

local sdu_xtmm2 =exp(_b[eq3:_cons])

return scalar sdu_xtmm2=`sdu_xtmm2'

local sdc_xtmm2 =exp(_b[eq4:_cons])

return scalar sdc_xtmm2=`sdc_xtmm2'

local sdn_xtmm2 =exp(_b[eq5:_cons])

return scalar sdn_xtmm2=`sdn_xtmm2'

local sdt_xtmm2 =exp(_b[eq6:_cons])

return scalar sdt_xtmm2=`sdt_xtmm2'

local landa_xtmm2=(`sdt_mm2'^2+ `sdu_xtmm2'^2)/(`sdc_mm2')^2

return scalar landa_xtmm2 = `landa_xtmm2'

return scalar niter_xtmm2=e(ic)

* local xtmm2_fail=_rc

* return scalar xtmm2_fail=_rc

}

}

end

**Stata code for fitting Mixture Models fitted using Stata’s Maximum Likelihood Routine**

*****

*** Mixture model MM**

*****

program define mm

version 9.2

args lnf theta1 theta2 theta3 theta4 theta5

quietly replace `lnf' = ln(exp(`theta5')*normd(($ML_y1-`theta1')/`theta3')/`theta3'+ normd(($ML_y1-`theta2')/`theta4')/`theta4' ) - ln(1+exp(`theta5')) if $ML_y2 ==0

quietly replace `lnf' = ln(normd(($ML_y1-`theta2')/`theta4')/`theta4' ) - ln(1+exp(`theta5')) if $ML_y2 ==1 & $ML_y3 ==0

quietly replace `lnf' = ln(exp(`theta5')* normd(($ML_y1-`theta1')/`theta3')/`theta3') - ln(1+exp(`theta5')) if $ML_y2 == 1 & $ML_y3 ==1

end

*****

*** Mixture model allowing for heteroscedasticity MM(Het)**

*****

program define mm_het

version 1.0

args lnf theta1 theta2 theta3 theta4 theta5 theta6

quietly replace `lnf' = ln(exp(`theta6')*normd(($ML_y1-`theta1')/`theta3')/`theta3'+ normd(($ML_y1-`theta2')/`theta4')/`theta4' ) - ln(1+exp(`theta6')) if $ML_y2 ==0

quietly replace `lnf' = ln(normd(($ML_y1-`theta2')/`theta4')/`theta4' ) - ln(1+exp(`theta6')) if $ML_y2 ==1 & $ML_y3 ==0

quietly replace `lnf' = ln(exp(`theta6')* normd(($ML_y1-`theta1')/`theta5')/`theta5') - ln(1+exp(`theta6')) if $ML_y2 == 1 & $ML_y3 ==1

end

*****

*** Mixture model with random effects RE-MM**

*****

program define re_mm

version 9

args todo b lnf g

tempvar rx tx mc mn lsc_u lsc_e lsn_e lor sc_u sc_e sn_e zn zc T S_z2 Sz_2 a t_lf gr1 gr2 gr3 gr4 gr5 gr6 last t1 t2

mleval `mc' = `b',eq(1)

mleval `mn' = `b',eq(2)

mleval `lsc_u' = `b',eq(3)

mleval `lsc_e' = `b',eq(4)

mleval `lsn_e' = `b',eq(5)

mleval `lor' = `b',eq(6)

quietly {

gen double `t_lf'=0

gen double `sc_u'=exp(`lsc_u')

gen double `sc_e'=exp(`lsc_e')

gen double `sn_e'=exp(`lsn_e')

local by $MY_panel

sort `by'

gen double `zc' = $ML_y1-`mc'

gen double `zn' = $ML_y1 -`mn'

gen `rx'= $ML_y2

gen `tx' =$ML_y3

sort `by'

by `by': gen `T' = _N

by `by':gen double `S_z2'=cond(_n==_N,sum(`zc'^2),.)

by `by':gen double `Sz_2'=cond(_n==_N,sum(`zc')^2,.)

by `by': gen `last' = _n==_N

gen double `a' =`sc_u'^2/(`T'*`sc_u'^2+`sc_e'^2)

*mixture model

replace `t_lf' = ln( exp(`lor')*normd(`zc'/`sc_e')/(`sc_e'*sqrt(2*c(pi))) ///

+ normd(`zn'/`sn_e')/(`sn_e'*sqrt(2*c(pi))) ) - ln(1+exp(`lor') ) if `rx' ==0

*linear model

replace `t_lf' = ln(normd(`zn'/`sn_e')/(`sn_e'*sqrt(2*c(pi)) ) )- ///

ln(1+exp(`lor')) if `rx' ==1 & `tx' ==0

* random effects model

replace `t_lf'=-.5*((`S_z2'-`a'*`Sz_2')/`sc_e'^2 + ln(`T'*`sc_u'^2/`sc_e'^2+1) + ///

`T'*ln(2*c(pi)*`sc_e'^2) ) + `T'*ln(exp(`lor')/(1+exp(`lor'))) ///

if `rx' == 1 & `tx' ==1 & `last'==1

mlsum `lnf'=`t_lf' if `last'==1

if (`todo'==0 | `lnf'>=.) exit

// compute the gradient

tempvar S_z

tempname d1 d2 d3 d4 d5 d6

gen double `gr1'=0

gen double `gr2'=0

gen double `gr3'=0

gen double `gr4'=0

gen double `gr5'=0

gen double `gr6'=0

*mixture model

gen double `t1'= exp(`lor')*normd(`zc'/`sc_e')/(`sc_e'*sqrt(2*c(pi)))

gen double `t2'= normd(`zn'/`sn_e')/(`sn_e'*sqrt(2*c(pi)))

replace `gr1'=`zc'*`t1'/ ((`t1'+`t2')*`sc_e'^2) if `rx' ==0

replace `gr2'=`zn'*`t2'/ ((`t1'+`t2')*`sn_e'^2) if `rx' ==0

replace `gr3'=0 if `rx' ==0

replace `gr4'=(`zc'^2-`sc_e'^2)*`t1'/((`t1'+`t2')*`sc_e'^2) if `rx' ==0

replace `gr5'=(`zn'^2-`sn_e'^2)*`t2'/((`t1'+`t2')*`sn_e'^2) if `rx' ==0

replace `gr6'= `t1'/(`t1'+`t2') -exp(`lor')/(1+exp(`lor')) if `rx' ==0

*linear model

replace `gr1' = 0 if `rx' ==1 & `tx' ==0

replace `gr2' = `zn'/(`sn_e'*`sn_e') if `rx' ==1 & `tx' ==0

replace `gr3' = 0 if `rx' ==1 & `tx' ==0

replace `gr4' = 0 if `rx' ==1 & `tx' ==0

replace `gr5' = `zn'*`zn'/(`sn_e'*`sn_e')-1 if `rx' ==1 & `tx' ==0

replace `gr6' = -exp(`lor')/(1+exp(`lor')) if `rx' ==1 & `tx' ==0

* random effects model

by `by': gen double `S_z' = sum(`zc')

by `by': replace `S_z' = `S_z'[_N]

replace `gr1' = (`zc'-`a'*`S_z')/`sc_e'^2 if `rx' == 1 & `tx' ==1

replace `gr2' = 0 if `rx' == 1 & `tx' ==1

replace `gr3' = `a'^2*`Sz_2'/`sc_u'^2 -`T'*`a' ///

if `rx' == 1 & `tx' ==1

replace `gr4' = `S_z2'/`sc_e'^2 - ///

`a'*`Sz_2'/`sc_e'^2 - ///

`a'^2*`Sz_2'/`sc_u'^2 - ///

`T'+1-`a'*`sc_e'^2/`sc_u'^2 ///

if `rx' == 1 & `tx' ==1

replace `gr5' = 0 if `rx' == 1 & `tx' ==1

replace `gr6' = `T'/(1+exp(`lor')) if `rx' == 1 & `tx' ==1

mlvecsum `lnf' `d1' = `gr1' , eq(1)

mlvecsum `lnf' `d2' = `gr2' , eq(2)

mlvecsum `lnf' `d3' = `gr3' if `last'==1 , eq(3)

mlvecsum `lnf' `d4' = `gr4' if `last'==1 , eq(4)

mlvecsum `lnf' `d5' = `gr5' if `last'==1 , eq(5)

mlvecsum `lnf' `d6' = `gr6' if `last'==1 , eq(6)

mat `g' = (`d1',`d2',`d3',`d4',`d5',`d6')

}

end

*****

*** Mixture model with random effects allowing**

*** for heteroscedasticity RE-MM(Het)**

*****

program define re_mm_het

version 9

args todo b lnf g

tempvar rx tx mc mn lst_u lsc_e lsn_e lst_e lor st_u sc_e sn_e st_e zn zc T S_z2 Sz_2 a t_lf gr1 gr2 gr3 gr4 gr5 gr6 gr7 last t1 t2

mleval `mc' = `b',eq(1)

mleval `mn' = `b',eq(2)

mleval `lst_u' = `b',eq(3)

mleval `lsc_e' = `b',eq(4)

mleval `lsn_e' = `b',eq(5)

mleval `lst_e' = `b',eq(6)

mleval `lor' = `b',eq(7)

quietly {

gen double `t_lf'=0

gen double `st_u'=exp(`lst_u')

gen double `sc_e'=exp(`lsc_e')

gen double `sn_e'=exp(`lsn_e')

gen double `st_e'=exp(`lst_e')

local by $MY_panel

sort `by'

gen double `zc' = $ML_y1 -`mc'

gen double `zn' = $ML_y1 -`mn'

gen `rx'= $ML_y2

gen `tx' =$ML_y3

sort `by'

by `by': gen `T' = _N

by `by':gen double `S_z2'=cond(_n==_N,sum(`zc'^2),.)

by `by':gen double `Sz_2'=cond(_n==_N,sum(`zc')^2,.)

by `by': gen `last' = _n==_N

gen double `a' =`st_u'^2/(`T'*`st_u'^2+`st_e'^2)

* noisily list `a' if _n > 750

*mixture model

replace `t_lf' = ln( exp(`lor')*normd(`zc'/`sc_e')/(`sc_e'*sqrt(2*c(pi))) ///

+ normd(`zn'/`sn_e')/(`sn_e'*sqrt(2*c(pi))) ) - ln(1+exp(`lor') ) if `rx' ==0

*linear model

replace `t_lf' = ln(normd(`zn'/`sn_e')/(`sn_e'*sqrt(2*c(pi)) ) )- ///

ln(1+exp(`lor')) if `rx' ==1 & `tx' ==0

* random effects model

replace `t_lf'=-.5*((`S_z2'-`a'*`Sz_2')/`st_e'^2 + ln(`T'*`st_u'^2/`st_e'^2+1) + ///

`T'*ln(2*c(pi)*`st_e'^2) ) + `T'*ln(exp(`lor')/(1+exp(`lor'))) ///

if `rx' == 1 & `tx' ==1 & `last'==1

mlsum `lnf'=`t_lf' if `last'==1

if (`todo'==0 | `lnf'>=.) exit

// compute the gradient

tempvar S_z

tempname d1 d2 d3 d4 d5 d6 d7

gen double `gr1'=0

gen double `gr2'=0

gen double `gr3'=0

gen double `gr4'=0

gen double `gr5'=0

gen double `gr6'=0

gen double `gr7'=0

*mixture model

gen double `t1'= exp(`lor')*normd(`zc'/`sc_e')/(`sc_e'*sqrt(2*c(pi)))

gen double `t2'= normd(`zn'/`sn_e')/(`sn_e'*sqrt(2*c(pi)))

replace `gr1'=`zc'*`t1'/ ((`t1'+`t2')*`sc_e'^2) if `rx' ==0

replace `gr2'=`zn'*`t2'/ ((`t1'+`t2')*`sn_e'^2) if `rx' ==0

replace `gr3'=0 if `rx' ==0

replace `gr4'=(`zc'^2-`sc_e'^2)*`t1'/((`t1'+`t2')*`sc_e'^2) if `rx' ==0

replace `gr5'=(`zn'^2-`sn_e'^2)*`t2'/((`t1'+`t2')*`sn_e'^2) if `rx' ==0

replace `gr6'=0 if `rx' ==0

replace `gr7'= `t1'/(`t1'+`t2') -exp(`lor')/(1+exp(`lor')) if `rx' ==0

*linear model

replace `gr1' = 0 if `rx' ==1 & `tx' ==0

replace `gr2' = `zn'/(`sn_e'*`sn_e') if `rx' ==1 & `tx' ==0

replace `gr3' = 0 if `rx' ==1 & `tx' ==0

replace `gr4' = 0 if `rx' ==1 & `tx' ==0

replace `gr5' = `zn'*`zn'/(`sn_e'*`sn_e')-1 if `rx' ==1 & `tx' ==0

replace `gr6' =0 if `rx' ==1 & `tx' ==0

replace `gr7' = -exp(`lor')/(1+exp(`lor')) if `rx' ==1 & `tx' ==0

* random effects model

by `by': gen double `S_z' = sum(`zc')

by `by': replace `S_z' = `S_z'[_N]

* replace `gr1' = 0 if `rx' ==1 & `tx' ==1

replace `gr1' = (`zc'-`a'*`S_z')/`st_e'^2 if `rx' == 1 & `tx' ==1

replace `gr2' = 0 if `rx' == 1 & `tx' ==1

replace `gr3' = `a'^2*`Sz_2'/`st_u'^2 -`T'*`a' ///

if `rx' == 1 & `tx' ==1

replace `gr4' = 0 if `rx' == 1 & `tx' ==1

replace `gr5' = 0 if `rx' == 1 & `tx' ==1

replace `gr6' = `S_z2'/`st_e'^2 - ///

`a'*`Sz_2'/`st_e'^2 - ///

`a'^2*`Sz_2'/`st_u'^2 - ///

`T'+1-`a'*`st_e'^2/`st_u'^2 ///

if `rx' == 1 & `tx' ==1

replace `gr7' = `T'/(1+exp(`lor')) if `rx' == 1 & `tx' ==1

mlvecsum `lnf' `d1' = `gr1' , eq(1)

mlvecsum `lnf' `d2' = `gr2' , eq(2)

mlvecsum `lnf' `d3' = `gr3' if `last'==1 , eq(3)

mlvecsum `lnf' `d4' = `gr4' if `last'==1 , eq(4)

mlvecsum `lnf' `d5' = `gr5' if `last'==1 , eq(5)

mlvecsum `lnf' `d6' = `gr6' if `last'==1 , eq(6)

mlvecsum `lnf' `d7' = `gr7' if `last'==1 , eq(7)

mat `g' = (`d1',`d2',`d3',`d4',`d5',`d6',`d7')

}

end
