# Supplementary material for: The implications of noncompliance for randomized trials with partial nesting due to group treatment
Source: Stat Med. 2020 Oct 28;40(2):349–68. doi: 10.1002/sim.8778 (PMC7821326; doi:10.1002/sim.8778)
Supplement: Supplementary file 3 — Data S3: Appendix 4 [file SIM-40-349-s003.docx]

| **Appendix 4** Data from lower back pain trial used for the analysis in table 2 | | | | | |
| --- | --- | --- | --- | --- | --- |
| Trial  ID | Randomisation | Group  Treatment  Received | Group ID | Visual Analogue  Scores | |
|  |  |  |  | Baseline | 9 Months |
| 1001 | Intervention | yes | 1 | 20 | 17 |
| 1002 | Control | no | 1002 | 48 | 19 |
| 1003 | Intervention | yes | 1 | 23 | 7 |
| 1004 | Intervention | no | 1004 | 26 | 6 |
| 1005 | Control | no | 1005 | 59 | 33 |
| 1006 | Intervention | yes | 1 | 51 | 33 |
| 1007 | Control | no | 1007 | 59 | 0 |
| 1008 | Control | no | 1008 | 58 | 0 |
| 1009 | Intervention | yes | 2 | 40 | 0 |
| 1010 | Intervention | no | 1010 | 50 | 50 |
| 1011 | Control | no | 1011 | 66 | 79 |
| 1012 | Control | no | 1012 | 78 | 30 |
| 1013 | Control | no | 1013 | 78 | 7 |
| 1015 | Intervention | yes | 2 | 35 | 25 |
| 1016 | Control | no | 1016 | 79 | 38 |
| 1017 | Control | no | 1017 | 29 | 6 |
| 1018 | Intervention | yes | 2 | 63 | 50 |
| 1019 | Intervention | no | 1019 | 27 | 26 |
| 1020 | Control | no | 1020 | 53 | 24 |
| 1021 | Intervention | no | 1021 | 20 | 0 |
| 1023 | Control | no | 1023 | 70 | 81 |
| 1024 | Intervention | yes | 1 | 55 | 56 |
| 1025 | Intervention | yes | 2 | 75 | 26 |
| 1026 | Control | no | 1026 | 47 | 48 |
| 1027 | Intervention | yes | 3 | 32 | 65 |
| 1028 | Control | no | 1028 | 46 | 40 |
| 1029 | Control | no | 1029 | 22 | 19 |
| 1030 | Intervention | yes | 2 | 50 | 13 |
| 1031 | Intervention | yes | 4 | 31 | 25 |
| 1032 | Control | no | 1032 | 42 | 51 |
| 1033 | Control | no | 1033 | 87 | 42 |
| 1035 | Control | no | 1035 | 28 | 8 |
| 1036 | Control | no | 1036 | 60 | 55 |
| 1037 | Intervention | yes | 4 | 22 | 3 |
| 1038 | Intervention | yes | 3 | 46 | 4 |
| 1039 | Control | no | 1039 | 52 | 54 |
| 1040 | Control | no | 1040 | 41 | 0 |
| 1042 | Intervention | yes | 3 | 27 | 4 |
| 1043 | Intervention | no | 1043 | 26 | 23 |
| 1045 | Intervention | yes | 5 | 30 | 3 |
| 1046 | Intervention | yes | 3 | 20 | 6 |
| 1047 | Control | no | 1047 | 33 | 8 |
| 1048 | Control | no | 1048 | 21 | 76 |
| 1049 | Intervention | no | 1049 | 52 | 31 |
| 1050 | Intervention | yes | 3 | 28 | 27 |
| 1051 | Intervention | no | 1051 | 48 | 82 |
| 1052 | Control | no | 1052 | 20 | 0 |
| 1053 | Intervention | yes | 5 | 30 | 30 |
| 1055 | Control | no | 1055 | 49 | 29 |
| 1057 | Control | no | 1057 | 20 | 0 |
| 1058 | Intervention | yes | 5 | 62 | 62 |
| 1059 | Intervention | yes | 5 | 42 | 20 |
| 1060 | Control | no | 1060 | 32 | 27 |
| 1061 | Intervention | yes | 7 | 42 | 19 |
| 1062 | Control | no | 1062 | 36 | 30 |
| 1063 | Control | no | 1063 | 50 | 18 |
| 1064 | Intervention | yes | 5 | 23 | 20 |
| 1066 | Intervention | yes | 6 | 73 | 52 |
| 1068 | Intervention | yes | 4 | 50 | 0 |
| 1069 | Control | no | 1069 | 59 | 12 |
| 1070 | Control | no | 1070 | 69 | 19 |
| 1072 | Intervention | yes | 5 | 38 | 18 |
| 1073 | Intervention | yes | 4 | 39 | 9 |
| 1074 | Control | no | 1074 | 46 | 0 |
| 1075 | Intervention | yes | 5 | 26 | 21 |
| 1076 | Intervention | no | 1076 | 21 | 4 |
| 1077 | Control | no | 1077 | 49 | 34 |
| 1078 | Control | no | 1078 | 23 | 2 |
| 1079 | Control | no | 1079 | 81 | 60 |
| 1080 | Intervention | no | 1080 | 60 | 9 |
| 1081 | Intervention | yes | 6 | 41 | 0 |
| 1082 | Control | no | 1082 | 37 | 7 |
| 1083 | Intervention | yes | 6 | 34 | 34 |
| 1084 | Control | no | 1084 | 67 | 15 |
| 1085 | Control | no | 1085 | 21 | 0 |
| 1086 | Control | no | 1086 | 38 | 47 |
| 1087 | Intervention | yes | 6 | 21 | 0 |
| 1090 | Intervention | no | 1090 | 86 | 36 |
| 1091 | Intervention | no | 1091 | 47 | 47 |
| 1092 | Intervention | yes | 7 | 35 | 18 |
| 1093 | Intervention | no | 1093 | 21 | 41 |
| 1094 | Control | no | 1094 | 91 | 18 |
| 1095 | Control | no | 1095 | 20 | 3 |
| 1096 | Intervention | no | 1096 | 56 | 40 |
| 1097 | Intervention | no | 1097 | 20 | 43 |
| 1098 | Intervention | yes | 7 | 26 | 20 |
| 1099 | Intervention | yes | 8 | 34 | 15 |
| 1100 | Intervention | yes | 9 | 26.5 | 76 |
| 1101 | Control | no | 1101 | 36 | 13 |
| 1102 | Control | no | 1102 | 63 | 38 |
| 1103 | Control | no | 1103 | 25 | 17 |
| 1105 | Intervention | yes | 7 | 50 | 31 |
| 1106 | Control | no | 1106 | 78 | 55 |
| 1108 | Control | no | 1108 | 82 | 88 |
| 1109 | Control | no | 1109 | 68 | 68 |
| 1110 | Intervention | yes | 8 | 28 | 6 |
| 1111 | Control | no | 1111 | 40 | 62 |
| 1112 | Intervention | yes | 7 | 56 | 36 |
| 1113 | Control | no | 1113 | 82 | 69 |
| 1114 | Intervention | yes | 9 | 54 | 34 |
| 1115 | Intervention | yes | 9 | 58 | 14 |
| 1116 | Control | no | 1116 | 76 | 69 |
| 1117 | Intervention | no | 1117 | 43 | 30 |
| 1118 | Control | no | 1118 | 38 | 50 |
| 1119 | Intervention | yes | 10 | 40 | 5.5 |
| 1120 | Control | no | 1120 | 66 | 73 |
| 1122 | Control | no | 1122 | 69 | 14 |
| 1123 | Intervention | yes | 8 | 38 | 12 |
| 1124 | Control | no | 1124 | 20 | 27 |
| 1126 | Intervention | no | 1126 | 85 | 63 |
| 1127 | Control | no | 1127 | 22 | 70 |
| 1128 | Control | no | 1128 | 28 | 41 |
| 1129 | Intervention | no | 1129 | 73 | 70 |
| 1130 | Intervention | yes | 9 | 37 | 44 |
| 1131 | Control | no | 1131 | 39 | 36 |
| 1132 | Intervention | yes | 12 | 50 | 0 |
| 1133 | Intervention | no | 1133 | 29 | 25 |
| 1134 | Intervention | yes | 10 | 35 | 12.5 |
| 1135 | Intervention | yes | 11 | 29 | 20 |
| 1136 | Control | no | 1136 | 37 | 27 |
| 1137 | Intervention | no | 1137 | 45 | 3 |
| 1138 | Intervention | yes | 12 | 71 | 39 |
| 1139 | Control | no | 1139 | 63 | 11 |
| 1140 | Control | no | 1140 | 70 | 75 |
| 1141 | Intervention | yes | 10 | 38 | 30 |
| 1142 | Control | no | 1142 | 42 | 3 |
| 1143 | Control | no | 1143 | 92 | 88 |
| 1144 | Intervention | no | 1144 | 97 | 81 |
| 1145 | Intervention | yes | 12 | 33 | 2 |
| 1146 | Intervention | yes | 13 | 75 | 0 |
| 1147 | Intervention | yes | 11 | 30 | 55 |
| 1148 | Control | no | 1148 | 73 | 10 |
| 1150 | Control | no | 1150 | 90 | 19 |
| 1152 | Control | no | 1152 | 36 | 21 |
| 1153 | Control | no | 1153 | 40 | 56 |
| 1155 | Intervention | no | 1155 | 39 | 23 |
| 1156 | Intervention | yes | 12 | 31 | 11 |
| 1157 | Intervention | yes | 10 | 66 | 54 |
| 1158 | Control | no | 1158 | 90 | 86 |
| 1159 | Intervention | no | 1159 | 43 | 87 |
| 1160 | Intervention | yes | 10 | 37 | 31 |
| 1161 | Control | no | 1161 | 73 | 50 |
| 1162 | Intervention | no | 1162 | 60 | 20 |
| 1163 | Intervention | yes | 11 | 81 | 52 |
| 1164 | Control | no | 1164 | 20 | 16 |
| 1166 | Control | no | 1166 | 36 | 3 |
| 1167 | Control | no | 1167 | 81 | 30 |
| 1169 | Control | no | 1169 | 95 | 99 |
| 1170 | Intervention | no | 1170 | 46 | 44 |
| 1171 | Control | no | 1171 | 26 | 53 |
| 1172 | Control | no | 1172 | 100 | 100 |
| 1173 | Control | no | 1173 | 86 | 65 |
| 1174 | Control | no | 1174 | 26 | 5 |
| 1175 | Control | no | 1175 | 22 | 2 |
| 1177 | Control | no | 1177 | 23 | 30 |
| 1178 | Intervention | no | 1178 | 41 | 30 |
| 1179 | Control | no | 1179 | 48 | 0 |
| 1181 | Control | no | 1181 | 45 | 54 |
| 1182 | Intervention | yes | 15 | 49 | 7 |
| 1183 | Intervention | no | 1183 | 57 | 13 |
| 1185 | Control | no | 1185 | 51 | 33 |
| 1186 | Intervention | yes | 13 | 23 | 4 |
| 1187 | Intervention | yes | 14 | 54 | 0 |
| 1188 | Control | no | 1188 | 65 | 14 |
| 1190 | Intervention | no | 1190 | 45 | 100 |
| 1191 | Intervention | yes | 14 | 32 | 3 |
| 1192 | Intervention | yes | 15 | 37 | 52 |
| 1193 | Intervention | no | 1193 | 52 | 40 |
| 1194 | Intervention | yes | 14 | 33 | 17 |
| 1195 | Intervention | yes | 15 | 51 | 35 |
| 1196 | Control | no | 1196 | 73 | 65 |
| 1198 | Intervention | no | 1198 | 67 | 65 |
| 1199 | Control | no | 1199 | 90 | 0 |
| 1200 | Intervention | yes | 15 | 37 | 3 |
| 1201 | Intervention | yes | 14 | 42 | 0 |
| 1202 | Control | no | 1202 | 52 | 33 |
| 1205 | Intervention | yes | 17 | 76 | 13 |
| 1206 | Control | no | 1206 | 24 | 4 |
| 1208 | Intervention | no | 1208 | 20 | 13 |
| 1209 | Intervention | yes | 14 | 39 | 74 |
| 1210 | Intervention | yes | 17 | 37 | 0 |
| 1211 | Control | no | 1211 | 69 | 26 |
| 1212 | Control | no | 1212 | 74 | 75 |
| 1213 | Intervention | yes | 16 | 34 | 0 |
| 1214 | Control | no | 1214 | 64 | 50 |
| 1215 | Control | no | 1215 | 37 | 66 |
| 1216 | Control | no | 1216 | 65 | 27 |
| 1217 | Intervention | yes | 17 | 38 | 0 |
| 1218 | Intervention | no | 1218 | 59 | 0 |
| 1219 | Intervention | yes | 16 | 59 | 35 |
| 1221 | Control | no | 1221 | 71 | 97 |
| 1222 | Intervention | no | 1222 | 89 | 50 |
| 1223 | Intervention | yes | 17 | 41 | 0 |
| 1224 | Control | no | 1224 | 61 | 5 |
| 1225 | Control | no | 1225 | 31 | 73 |
| 1226 | Intervention | no | 1226 | 27 | 14 |
| 1228 | Control | no | 1228 | 32 | 31 |
| 1229 | Control | no | 1229 | 51 | 79 |
| 1230 | Intervention | yes | 16 | 35 | 23 |
| 1231 | Intervention | no | 1231 | 33 | 0 |
| 1232 | Control | no | 1232 | 20 | 13 |
| 1233 | Control | no | 1233 | 66 | 7 |
| 1234 | Control | no | 1234 | 30 | 4 |
